# The morphogenetic activity of dAnk genes in the diatom *Thalassiosira pseudonana* is sensitive to Si availability

**DOI:** 10.1038/s42003-026-10583-4

**Published:** 2026-06-29

**Authors:** Lior Aram, Neta Varsano, Olga Brontvein, Smadar Levin-Zaidman, Christoph Heintze, Assaf Gal

**Affiliations:** 1 Department of Plant and Environmental Sciences, Weizmann Institute of Science, Rehovot, Israel; 2 Department of Chemical Research Support, Weizmann Institute of Science, Rehovot, Israel; 3 B CUBE – Center for Molecular Bioengineering, Technische Universität Dresden, Dresden, Germany

**Keywords:** Element cycles, 3-D reconstruction, Cryoelectron tomography

## Abstract

The intricate morphologies of the silica cell walls of diatoms are genetically controlled, as each species has its own unique ornamentation. The process of diatom silicification, from the initial uptake of soluble silicate from the environment, through the cellular deposition and morphogenesis of the mature silica element, is orchestrated by an array of macromolecules. It was recently shown that dAnk proteins, which are involved in silica formation, are regulators of the silica pore pattern. In this work, we study the influence of another controller, silicate availability in the environment, on silica phenotype in dAnk knockout mutants. Using various microscopy techniques, we show that the phenotype of some mutants is variable, ranging from large gaps in the silica to pore-less silica coverage. In situ cryo-electron tomography shows that not only is morphogenesis affected, but also the regulation of the silica deposition vesicle is altered. These results point to an additional role of the dAnk proteins, where they are involved in the regulation of intracellular silicification, and when their expression is disrupted, silicification becomes sensitive to silicate availability. This points to synergistic regulation of silicification, where the function of a specific protein is context-dependent.

## Abstract (web summary)

Knock-out mutants of diatom dAnk genes reveal that intracellular silicification is synergistically regulated by dAnk proteins and environmental silicate availability.

## Introduction

Diatoms are highly abundant and morphologically diverse unicellular algae distinguished by forming a silicified cell-wall composed of several silica elements^1,2^. The formation of each silica element occurs intracellularly in a silica deposition vesicle (SDV)^3^, and upon completion, the whole structure is exocytosed^4^. The silicification process is under tight biological regulation, as each diatom species is characterized by specific architecture and ornaments of its cell wall^5–9^. Although many silica-associated proteins and other macromolecules were studied, the precise molecular mechanisms governing the regulation of silica deposition and morphogenesis remain unknown^10^.

The first proteins related to silica formation in diatoms were discovered by their isolation from extracted silica elements^11–15^. Several families of proteins that are either embedded within the silica or associated with its surface were documented^16–19^. In recent years, more proteins have been reported to be associated with silica formation without direct contact with the silica itself. These include SDV membrane proteins integrated into the SDV membrane^20–22^, and putative silicate transporters involved in the silicate transport process^23,24^. A recent addition to the portfolio of genes implicated in silicification is the *dAnk* gene family^25^, which includes three members identified in the genome of the model diatom species *Thalassiosira pseudonana* (recently reclassified as *Cyclotella nana*). The *dAnk* proteins are characterized by the presence of ankyrin repeat domains and are cytoplasmic^25^. Based on their localization, it has been hypothesized that they may interact with SDV-associated proteins from the cytoplasmic side.

Targeted knockout of *dAnk* genes in *T. pseudonana* led to distinct morphological alterations in the pore pattern of the silica valve, the lid-like element located at both ends of the barrel-shaped cell^25^. Each single *dAnk* gene deletion influenced the nanometer-scale pores, with notable differences in pore organization and density relative to the wild-type. Despite these phenotypic effects, the molecular function of the *dAnk* proteins remains uncharacterized, and their mechanistic role in regulating silica morphogenesis is yet to be determined.

A crucial aspect in diatom silicification is their ability to uptake soluble silicate species from their environment (thereafter referred to as Si), concentrate them intracellularly, and translocate them into the SDV, where polymerization into solid silica occurs^26–30^. Investigating the effects of external Si concentrations on intracellular silicification processes is challenging, as almost all diatoms arrest their cell cycle under Si-limiting conditions^31,32^. Practically, cell wall morphology remains largely unchanged across a wide range of Si concentrations until a critical minimal threshold is reached, below which silicification ceases. This inherent feature of Si-dependent cell cycle arrest is commonly exploited in experimental protocols to synchronize silica formation in cultures by allowing cells to recover from Si deprivation^31^. In wild-type cells, silica morphology is not affected by limiting Si conditions, and once Si is above the minimal threshold, the cells silicify in accordance with the same regulated morphology.

In this work, we studied how Si limitation affects silica morphologies in *T. pseudonana dAnk* knockout mutants. We examined six distinct genetic backgrounds: three single knockouts targeting *dAnk1*, *dAnk2*, and *dAnk3*^25^, and three double knockout combinations of these genes. Using electron microscopy, we show that the mutant exhibiting the lowest pore density under Si-replete conditions, the *dAnk1/3* double knockout strain (*dAnk1/3dKO*), exhibits a markedly altered phenotype at Si-deplete conditions. This altered phenotype is characterized by large gaps in the silica valves and abnormal formation of SDVs, leading to the deposition of multiple silica layers within the valve structure.

## Results

In this work, we studied the morphological characteristics of valves in the six mutant strains. Four of these mutants have been previously reported^25^, including three single knockouts in *dAnk1KO*, *dAnk2KO*, and *dAnk3KO*, and one double knockout *dAnk1/3dKO*. The remaining two mutants, *dAnk1/2dKO* and *dAnk2/3dKO*, were generated for this study using the same approach. Genomic PCR confirmed the presence of frameshift mutations in all six strains, resulting in truncated polypeptides (Figs. S1, S2).

**Fig. 1:**
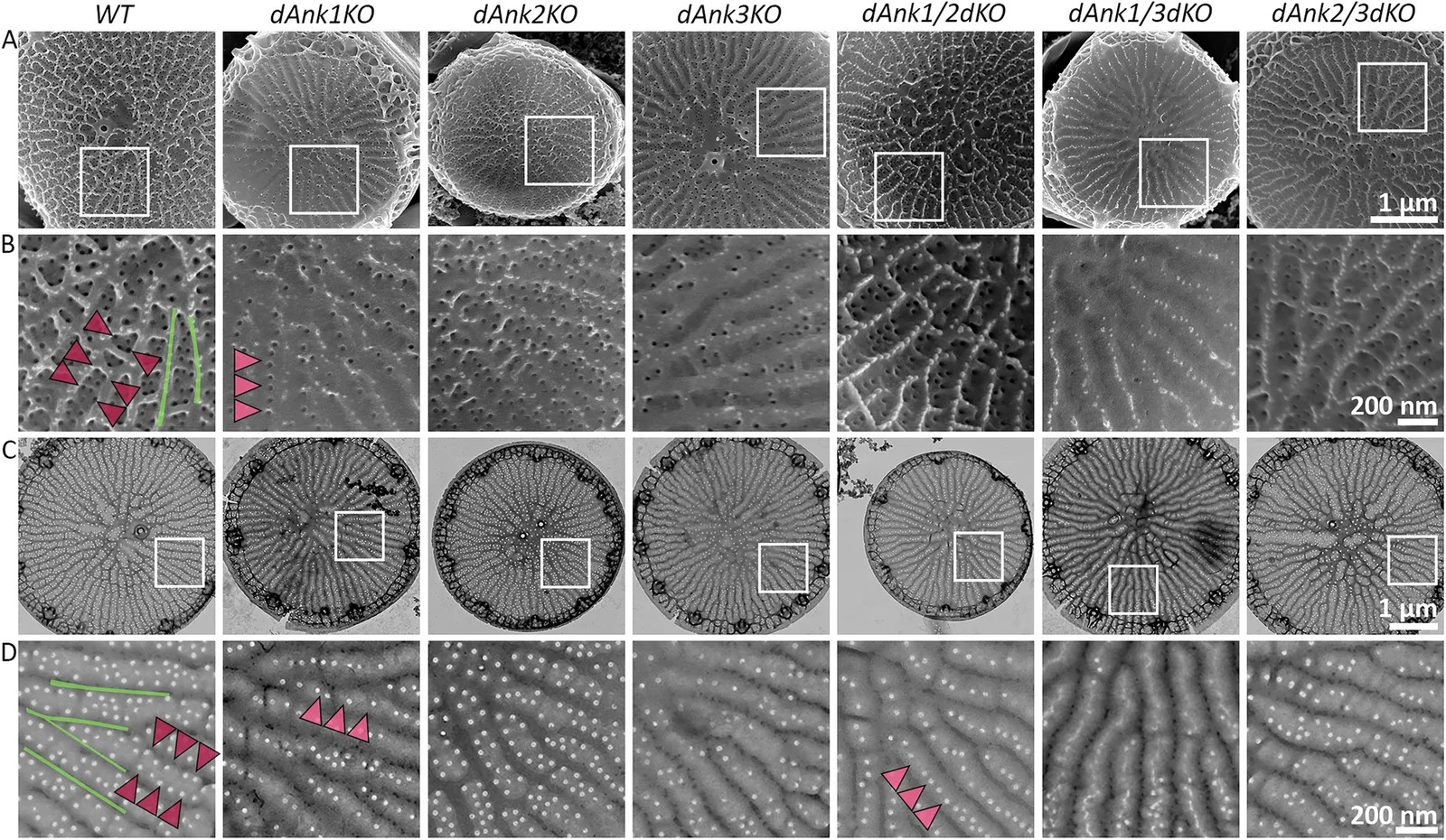
The valve silica morphology of the six *dAnk* knockout strains. **A**, **B** SEM images of the valves. **C**, **D** TEM images of extracted valves. **B** and **D** are high magnifications of the squares in A and C. Ribs are indicated in green. Pink and light pink arrowheads indicate two or single lines of nanopores arranged between two ribs, respectively.

Valves from these six *dAnk* knockout mutants were imaged using scanning electron microscopy (SEM) and transmission electron microscopy (TEM) under normal, Si-replete conditions (Fig. 1). In wild-type cells, *T. pseudonana* valves exhibit a circular morphology, defined by thick silica ribs radiating from the central region. These ribs are connected by a thin silica layer containing regularly arranged circular nanopores, known as cribrum pores, approximately 30 nm in diameter. The previously reported mutant strains demonstrate their identified phenotypes^25^, in the *dAnk1KO* strain, the nanopores show an abnormal spatial distribution, clustering centrally between adjacent ribs rather than aligning alongside the ribs as seen in wild-type cells. In contrast, *dAnk2KO* mutants exhibited an increase in pore density, whereas *dAnk3KO* strains showed a reduction in pore number (Fig.1). These phenotypes became more pronounced in the double knockouts. The new *dAnk1/2dKO* strain exhibited a mosaic valve pattern, reminiscent of the *dAnk1KO* phenotype, including patches of fewer nanopores that align to the center between two ribs. The new *dAnk2/3dKO* mutants showed fewer circular nanopores and disrupted rib organization. Notably, the already reported *dAnk1/3dKO* displayed the most severe phenotype, with valves exhibiting a pronounced reduction in nanopore number and size (Fig. 1 and S3). In summary, the knockout of dAnk genes results in alterations to valve nanopore patterning, with pronounced effects observed in double mutants.

To investigate Si limiting conditions, we maintained the cultures for 72 hours in 20 µM Si medium (Si-deplete), with daily dilutions into fresh medium to maintain relatively constant Si concentrations (Table S1). Under these conditions, cells exhibited an average of two population doublings per 24 hours, resulting in approximately 95% of the population being produced within the 72-hour period (Fig. S4 and Table S1). An experiment with the Si-replete 330 µM Si was conducted as a control, showing a slightly faster growth rate for all strains (Fig. S4).

**Fig. 2:**
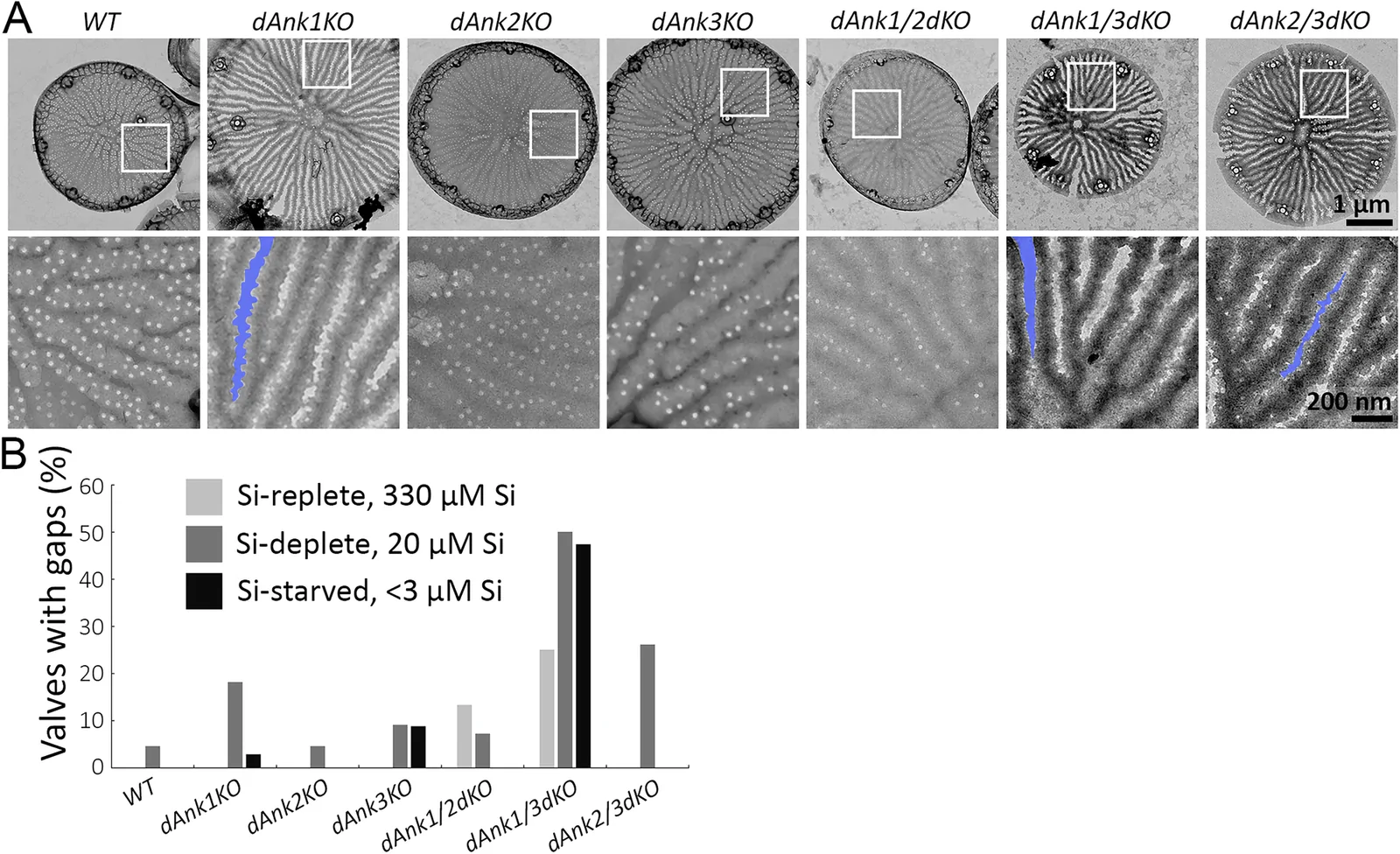
Elongated gaps appear under Si limiting conditions. **A** TEM images of extracted valves of the six independent *dAnk* knock out strains. Blue annotations – elongated gaps. **B** Analysis of the percentage of valves containing elongated gaps of the different strains, under the three different growth conditions (Si-starved experiments are presented later in the text). The number of valves counted for each condition is provided in the Source data.

We collected TEM images of ~20 valves from each strain to evaluate the Si-deplete phenotype. Remarkably, a notable phenotype in Si-deplete conditions was elongated gaps between silica ribs (Fig. 2A). These gaps, measuring between 50 and 200 nanometers in width (Fig. S5), were observed in only about 5% of wild-type cells but appeared in approximately 50% of *dAnk1/3dKO* valves (Fig. 2B). This striking phenotype was consistently observed across two additional and genetically independent *dAnk1/3dKO* lines (Fig. S6). Moreover, elongated gaps were detected in ~25% of *dAnk1KO* and *dAnk2/3dKO* valves. In comparison, other mutant valves showed this morphology in fewer than 10% of the valves. Notably, the *dAnk1/3dKO*, characterized by the lowest nanopore density at Si-replete conditions, demonstrated the most dramatic sensitivity to silicate availability, with a correspondingly high frequency of elongated gaps between ribs.

**Fig. 3:**
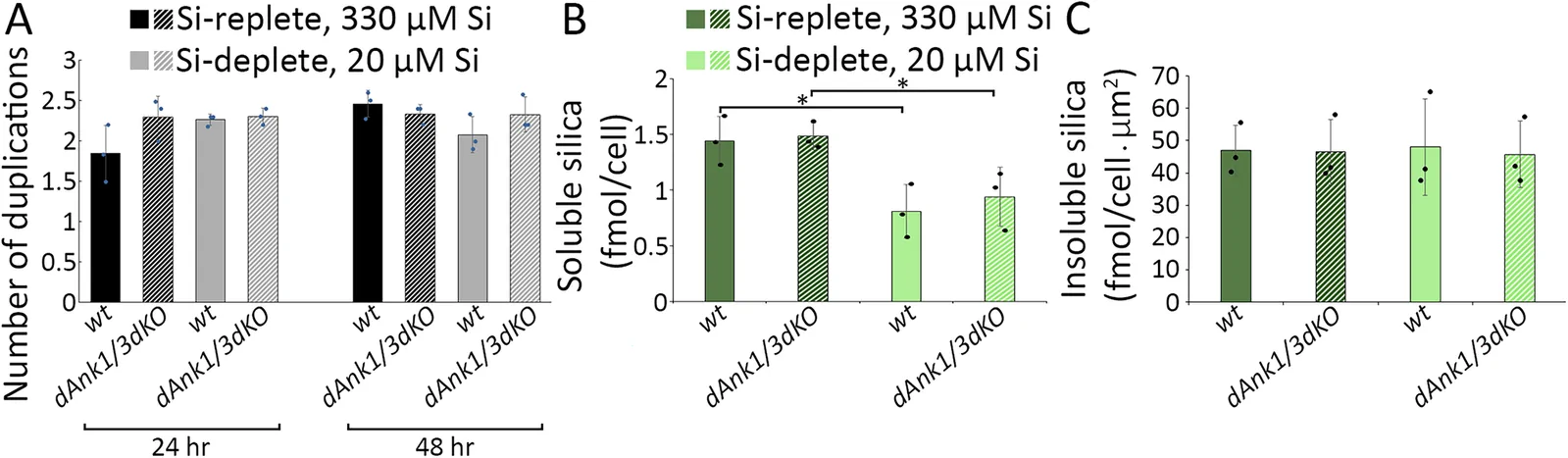
Cellular viability and silica content in *dAnk1/3dKO* under Si-deplete conditions. Data are presented as the average of three independent biological experiments ± standard deviation. **A** The calculated number of WT and *dAnk1/3dKO* cell duplications under 330 µM or 20 µM Si for 24 and 48 hours. The amount of soluble silica (**B**) or insoluble silica (**C**) per cell in conditions of 330 µM or 20 µM Si for 48 hours. One-way ANOVA test shows no significant differences between the number of duplications of wt and dAnk1/3 after 24 hours (*p* = 0.103) or 48 hours (*p* = 0.165), and in insoluble silica between wt and dAnk1/3 (*p* = 0.995) in the two different Si concentrations. One-way ANOVA with t-test shows significant differences in soluble silica between wt in replete and deplete Si conditions (*p* = 0.028*) and in soluble silica between dAnk1/3 in replete and deplete Si conditions (*p* = 0.032*). No significant differences between wt and dAnk1/3 in replete medium (*p* = 0.799), and in deplete medium (*p* = 0.561) (Table S2).

To investigate whether the elongated gap phenotype observed in the *dAnk1/3dKO* affects cell viability, we monitored the growth rates of the *dAnk1/3dKO* and wild-type strains with daily resolution under both Si conditions. The growth rates revealed no statistically significant differences between the WT and *dAnk1/3dKO* cells in either condition (Fig. 3A), indicating that *dAnk* knockout does not impair overall cell proliferation. To investigate whether the elongated gap phenotype is associated with altered intracellular silica pools, we quantified both soluble and insoluble Si fractions in the cultures. To quantify soluble silica, cells were first boiled, and the Si concentration in the resulting supernatant was then measured. To quantify insoluble silica, the pellet separated from the boiled cells was dissolved in NaOH, followed by acidification with HCl, and Si concentration was measured^29^. No significant differences were observed between the WT and *dAnk1/3dKO* cells in either the soluble (Fig. 3B) or insoluble (Fig. 3C) Si content. The concentration of insoluble silica remained consistent across both treatments. However, the levels of soluble Si were lower in cultures grown under Si-deplete conditions. These findings suggest that *dAnk1/3dKO* does not affect silica uptake, production, or cell viability, even in Si-deplete conditions that affect the level of intracellular soluble Si. This means that silica absent from the mutant gaps is compensated in other locations, such as thicker ribs.

In a second experimental approach, similar to the known cell-cycle synchronization protocols^24^, cells were transferred to a silicate-deprived medium in which cell-cycle is arrested for 16 hours. After this acute silicate starvation period, silicate was reintroduced at a concentration of 330 µM Si, so the cells started to silicify. PDMPO, a fluorophore that incorporates specifically into newly synthesized silica, was used to localize valves that formed after the Si-starvation period (Fig. S7). After a 3-hour recovery period, approximately 50% of the cells exhibited silicification activity (detected as PDMPO fluorescence). TEM images of PDMO-stained isolated valves showed the elongated gaps between silica ribs as observed in the long-term cultivation (Fig. 2B and Fig. S8). Altogether, these findings reveal a link between the function of dAnk proteins in silica morphogenesis and conditions of environmental silicate levels.

**Fig. 4:**
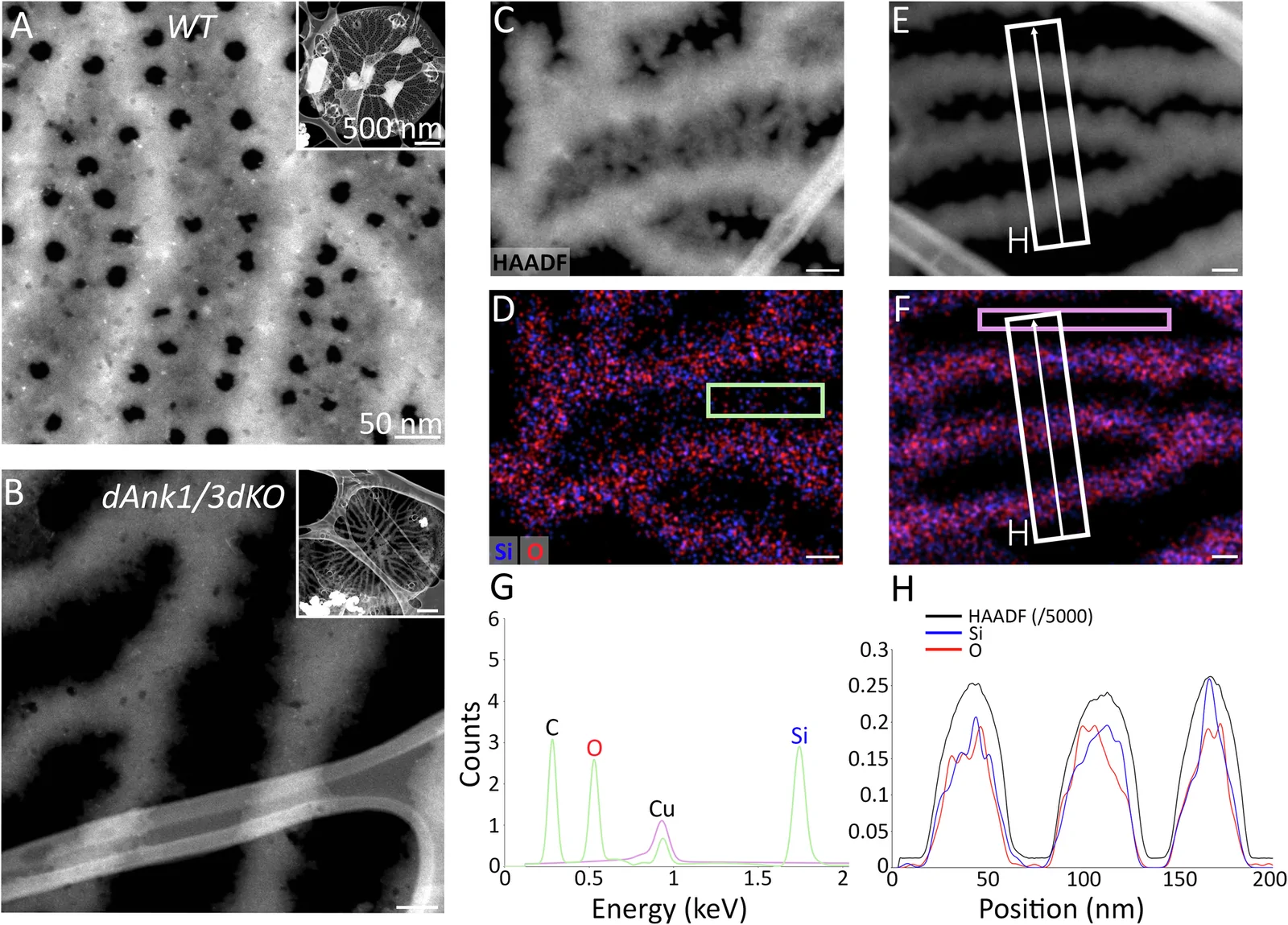
High-resolution investigation of *dAnk1/3dKO* valve formed at Si-deplete conditions. **A**, **B** HAADF-STEM images of the wild type and *dAnk1/3dKO* valves. Insets show the whole valve. HAADF-STEM images of *dAnk1/3dKO* valves showing areas where silica is present between the ribs (**C**), and areas of gaps between the ribs (**E**). **D**, **F** EDS maps of **C** and **E**, respectively, show the localization of silicon and oxygen. **G** EDS spectrum of the green (**D** – where thin silica is present) and pink (**F** – an empty gap) areas in (**D**) and (**F**). **H** EDS spectrum of the white line in F shows silicon, oxygen, and HADDF (HAADF values were divided by 5000 for clarity).

To characterize the gap phenotype observed in the *dAnk1/3dKO* valves, we used Scanning Transmission Electron Microscopy coupled with Energy-Dispersive X-ray Spectroscopy (STEM-EDS). To maintain a constant silicate concentration for seven days, wild-type and *dAnk1/3dKO* cells were cultured in semi-permeable plastic inserts positioned above a high-volume reservoir of 10 µM Si medium. Valves of wild-type or *dAnk1/3dKO* cells grown under these conditions were imaged (Fig. 4A, B). The micrographs reveal prominent empty regions between the ribs in the *dAnk1/3dKO* valves. To determine whether the inter-rib gaps in the *dAnk1/3dKO* valves were truly devoid of material or occupied by an ultra-thin silica layer, we conducted EDS spectral analysis (Fig. 4C–H). Comparative elemental mapping of the rib-adjacent regions confirmed that the elongated gaps lack detectable silica, indicating they are indeed empty.

**Fig. 5:**
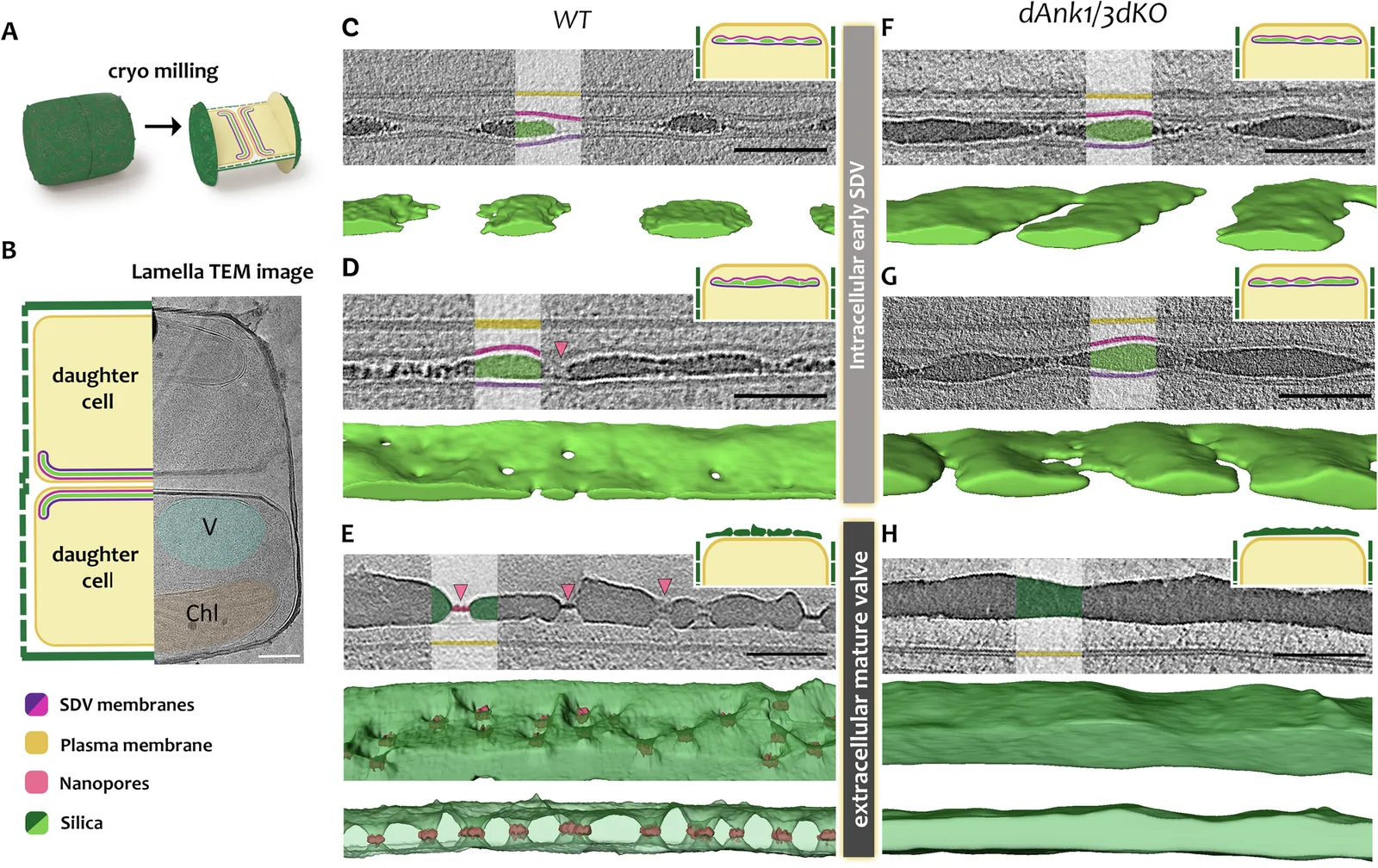
Imaging wild-type and *dAnk1/3dKO* valves in situ with cryoET. **A** Scheme of a cell before and after lamella preparation. **B** A cryo-TEM image of a lamella in a dividing cell that contains SDVs. The scheme on the left and the colors on the lamella image show the different organelles (V-vacuole, Chl-chloroplast). The scale bar is 500 nm. **C**, **D**, **F**, **G** A slice through the reconstructed volume of an SDV is shown above a surface representation of the segmented silica of wild-type and *dAnk1/3dKO*. **E**, **H** A slice through the reconstructed volume of a mature valve above a surface representation of the silica with nanopores (wild-type) or without the nanopores (*dAnk1/3dKO*). Scale bars in **C**–**F** are 100 nm. Datasets shown in (**C**–**E**) were previously reported^34^.

In the next step, we aimed to investigate the process of silica formation within the silica deposition vesicle (SDV) of cells that recover from Si starvation, to evaluate what are the structural differences compared to the process at Si-replete conditions. We used cryo-electron tomography (cryo-ET), which enables high-resolution visualization of cellular structures in their native state^4,33,34^. A culture synchronized by the acute Si starvation treatment was allowed to recover for 3 hours to initiate active silicification. At this stage, cells were vitrified and subsequently thinned to ~200 nm lamellae using a cryo-focused ion beam (cryo-FIB), enabling acquisition of tomographic datasets of the silicification process (Fig. 5A, B). We acquired 32 high-resolution cryo-ET datasets of dividing cells, imaging SDVs, and post-exocytosis valves (Fig. S9).

Consistent with previous observations^34^, the early stages of valve formation in wild-type cells are characterized by the lateral growth of silica ribs. Subsequently, the inter-rib spaces were filled with thinner silica, and nanopores began to form (Fig. 5C, D). In mature valves following exocytosis, the nanopores are filled with a material of unclear composition (Fig. 5E). In contrast, while the early stages of valve formation in the *dAnk1/3dKO* mutant resembled those of the wild-type (Fig. 5F, G), mature valves that formed before Si-starvation were characterized by the absence of nanopores (Fig. 5H), same phenotype that was observed by imaging mature extracted valves.

**Fig. 6:**
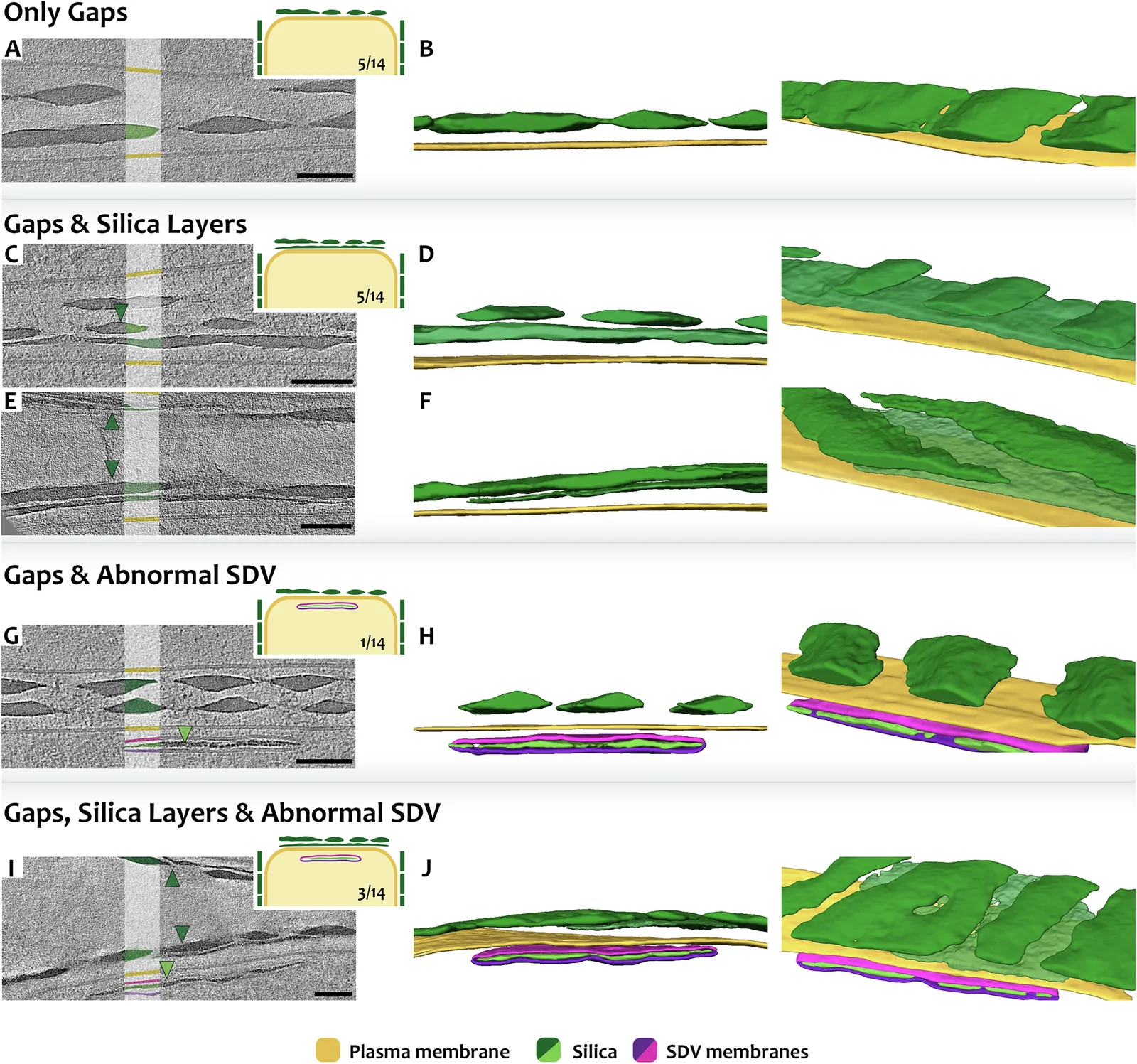
Structural abnormalities in valve formation of *dAnk1/3dKO* recovering from Si-starved conditions. We divided the observed phenotypes into four categories. In each, a slice through the reconstructed volume of an SDV is shown. Insets show schematic representations of the cell state. Each dataset contains regions from two daughter cells, in the middle and right columns, surface representations that relate only to the lower cell in each dataset are presented for the segmented silica, SDV membranes, and plasma membrane. Numbers indicate the counts of datasets displaying each specific phenotype relative to the total acquired dataset, which have newly formed valves. Scale bars are 100 nm.

We analyzed 23 cryo-ET datasets collected from the *dAnk1/3dKO* cells following valve exocytosis and prior to sister cell separation (Fig. 6), therefore representing the mature state of recently formed valves. 14 datasets displayed valves with pronounced discontinuities in their silica architecture, characterized by prominent inter-rib gaps, whereas the other nine valves had no nanopores or very few nanopores. This proportion is consistent with the ~50% of mature valves exhibiting elongated gaps observed in extracted valves under Si-deplete and Si-starved conditions (Fig. 2B).

Notably, cryo-ET analysis further revealed that these inter-rib gaps were frequently accompanied by additional ultrastructural abnormalities. Two recurring features were observed (sometimes apart and sometimes in the same cell): (i) the presence of small SDVs, which appeared in four of the datasets (Fig. 6G–J), and (ii) the formation of an additional silica layer, which appeared in eight of the datasets (Fig. 6C–F, I, J). The abnormal SDVs were smaller in diameter than the cell diameter and contained a thin, immature silica layer. These SDVs were observed in the cytoplasm concomitant with the exocytosed valve, suggesting that they formed subsequent to the exocytosis of the primary, mature SDV. The second structural abnormality, the additional silica layer, appeared as a thin silica layer located in close proximity to the main valve, often nearly in contact with it. The additional silica layers exhibited fewer gaps and appeared continuous. In approximately three of the datasets, the thin silica layer co-occurred with the abnormal SDVs. These findings suggest that dAnk proteins are involved in multiple aspects of silica deposition and frustule morphogenesis. Moreover, the manifestation of these defects under Si-limiting conditions indicates that dAnk function is modulated by Si availability.

## Discussion

In this study, we investigated the interrelations between Si availability and silica morphogenesis in *dAnk* knockout mutants. We found that the phenotype of some mutant strains, most notably the *dAnk1/3dKO*, displays a Si-dependent morphology of the valves, with Si-deplete conditions leading to gaps between the silica ribs and Si-replete conditions yielding pore-less silica coverage. High-resolution cryo-ET further revealed that SDV formation in this mutant is also perturbed, with abnormal SDVs that form double or even triple layers of silica. These results widen the scope of current interpretations of the activity of the dAnk proteins beyond the initial observations on their effect on pore patterning^25^. It is evident that the altered pore phenotype is dependent of other factors such as silicate availability, pointing to a role for a possible relation of the dAnk proteins to regulation of silica transport into the SDV, even though they do not directly alter cellular Si content and viability. In that case, it can be extrapolated that the varied density of pores in the *dAnk* mutants, even at Si-replete conditions, might be related to dysregulated Si transport.

The current model for dAnk function suggests the presence of transient nanodroplets within the SDV, which are regulated by the dAnk proteins and template the nanopores^25^. Therefore, the concomitant loss of nanopores and appearance of elongated gaps under Si limitation might be the result of nanodroplet coalescence that yields extended, elongated domains under Si limitation rather than discrete pore templates. Consistent with this view, the dAnk knockouts do not perturb silica rib formation, suggesting that dAnk proteins primarily influence pore templating rather than rib growth. In the future, the continued effort to understand the function of dAnk protein should take into account that its effect can be context specific, meaning that Si availability and transport give rise to different cellular responses, which are also manifested in pore patterning.

The fact that the morphogenetic phenotype is variable between mutants and even within different cells of the same strain highlights several points. First, it is not surprising to find redundancy in the function of different members of the same gene family, and a triple *dAnk* mutant was not generated. Moreover, diatoms can silicify in extremely low Si concentrations, and the 20 µM Si that was used as a Si-limiting concentration was probably sufficient for some ‘regular’ silicification in part of the cell population before it was further depleted^29^. In this context, it is interesting that the cells recovering from Si-starvation showed the same phenotype in the first hours of stress recovery as cells grown in continuous Si-limitation (Fig. 2B). This may point to a cellular cascade that is activated by Si-limitation and leads to the formation of the gaps. Even after alleviation of the stress and resuming the cell cycle, there is still a lag phase that is manifested in the low-Si phenotype even after the supplementation of ample Si^28^.

Our cryo-ET data show that under Si limitation, abnormal SDVs are present in the *dAnk1/3dKO*. The observed SDVs appear smaller than the cell diameter and correlate with fragmented, newly formed valves. These abnormal SDVs can be the initial step in the formation of the thin silica sheets, which, after exocytosis, are detected as layers juxtaposed to the newly formed valve in dividing *dAnk1/3dKO* cells grown under Si limitation (approximately half of the observed cells had abnormal silica layers inside or outside the cell, Fig. 6C–J). Given that the *dAnk* double knockout grows at a comparable rate to the wild type under Si limitation (Fig. 3A), these observations raise questions about the functional relationship between silicification and cell-cycle progression in diatoms. Nevertheless, currently, there is not enough information to substantiate a comprehensive mechanism that will link all the observations, and further genetic and structural studies are needed.

## Methods

### Cell cultures

*Thalassiosira pseudonana* (CCMP1335) cultures were grown in filtered natural Mediterranean seawater adjusted to 3.5% salinity and supplemented with the f/2 nutrient recipe and 330 µM silicic acid. Cultures were maintained at 18 °C under 16/8 h light/dark cycles.

### Construction of plasmids for the dAnk gene knockout

The dAnk2KO construct for double knockout was generated as follows: The nourseothricin resistance gene of the plasmid pTpKO/dAnk2 was exchanged with the blasticidin resistance gene. Therefore, pTpKO/dAnk2 and pTpfcp/BLAST were digested with BamHI. The plasmid backbone of pTpKO/dAnk2 and the blasticidin resistance gene were gel-purified and ligated. The final plasmid is termed pTpfcpBLAST/TpKO/dAnk2.

### Generation of dAnk double mutants

The dAnk1/2 knockout strain was generated by introducing the pTpfcpBLAST/TpKO/dAnk2 plasmid into the dAnk1KO3 single knockout strain. The dAnk2/3 knockout strain was generated by introducing the pTpfcpBLAST/TpKO/dAnk3 plasmid^25^, into the dAnk2KO1 single knockout strain. The biolistic transformation of *T. pseudonana* and the screening for gene knockout mutants were performed as described previously.

### Si-deplete culture maintenance

Exponentially growing cultures were diluted in filtered seawater (FSW) containing either 20 µM Si (deplete) or 330 µM Si (replete) to a final concentration of 1 × 10⁵ cells mL⁻¹ in a total volume of 10 mL. After 24 hours, cell concentrations were measured using a Z2 Coulter counter, and cultures were again diluted with the corresponding medium (20 or 330 µM Si) to restore a concentration of 1 × 10⁵ cells mL⁻¹ in 10 mL. This dilution procedure was repeated twice to maintain cells in exponential growth under the designated silicon conditions.

### Silica starvation and culture synchronization

Synchronization of the six *dAnk* knockout mutant strains was performed using the Si starvation protocol, as previously described^34^. Cultures were maintained in exponential growth phase under 12 h light/12 h dark cycles by diluting (1:10) into fresh medium every other day during the week preceding synchronization. To induce Si starvation, 100 ml aliquots of culture were centrifuged at 3000 × *g* for 10 min and resuspended in filtered seawater (with < 3 μM Si). This washing step was repeated three times. The cultures (~0.5 million cells/ml) were then grown in the dark for 12 h with agitation in a Si-free medium. Cultures were subsequently transferred to continuous light and maintained under Si-starvation conditions for an additional 4 h. Cell cycle arrest was verified by monitoring cell density which increased by less than 20% during prolonged incubation under these conditions. At the end of the Si starvation period, cells were concentrated to approximately 4 million cells/ml, and Si was replenished to 330 µM. PDMPO [2-(4-pyridyl)−5-((4-(2-dimethylaminoethyl-amino-carbamoyl)methoxy)-phenyl)oxazole] (Thermo Fisher Scientific, Waltham, MA, USA) was added to label newly formed silica. PDMPO fluorescence was monitored by imaging the cultures with an epifluorescence microscope (Nikon Eclipse Ni-U, ex: 365 nm, em: 525 nm). After 3 hours from Si addition, the cells were subjected to the sodium hypochlorite valve extraction protocol or underwent cryo-fixation using plunge freezing.

### Sodium hypochlorite-based sample preparation for SEM and TEM

Cell cultures were pelleted by centrifugation at 3000 × *g* for 5 min, and washed twice with Milli-Q water. Cells were then resuspended in 0.06% sodium hypochlorite and incubated at 4 °C for approximately 6 h with agitation. The sodium hypochlorite solution was subsequently replaced with a fresh solution, and incubation was continued for approximately 16 h at 4 °C with agitation. Following treatment, cells were washed with Milli-Q water and prepared for imaging. For SEM, samples were pipetted onto track-etched membranes (Sigma-Aldrich, St. Louis, MO, USA) and mounted on conductive carbon tape attached to aluminum stubs. For TEM, samples were applied to 200-mesh copper grids, and excess water was removed by blotting with Whatman paper.

### SEM imaging

Samples were sputter-coated with 3.5 nm Iridium (Safematic GmbH, Zizers, Switzerland) and imaged using a Sigma 500 scanning electron microscope (Zeiss, Oberkochen, Germany), with an accelerating voltage of 5 kV, an aperture size of 20 μm, and a working distance of approximately 3–5 mm.

### TEM imaging

The TEM samples were imaged using a Titan Krios G3i TEM (Thermo Fisher Scientific, Waltham, MA, USA), operating at 300 kV. Each image is a montage of images taken in counting mode at a nominal magnification of 26,000x, corresponding to a physical pixel size of 0.34 nm. Some TEM images were taken in the Tecnai Spirit transmission electron microscope (FEI, Eindhoven, Netherlands) operated at 120 kV.

### Measurement of intracellular soluble and insoluble silica fractions

The soluble and insoluble silica contents in wild-type and *dAnk1/3dKO* cells were quantified following an established protocol^29^, in order to investigate if there is a difference between the various strains. Exponentially growing cultures were diluted two days prior to the experiment in filtered seawater (FSW) medium to a concentration of 2 × 10⁵ cells mL⁻¹. Approximately 3 × 10⁷ cells were collected by centrifugation at 16,500 × *g* for 4 min and washed twice with Si-free medium. The resulting pellet was resuspended in 1 mL of Milli-Q water and boiled for 10 min. After cooling to room temperature, the samples were centrifuged again at 16,500 × *g* for 4 minutes. The supernatants were collected and analyzed for intracellular soluble silica using a silicate test kit (Merck Millipore, USA) according to the manufacturer’s instructions. The remaining pellets (insoluble fraction) were resuspended in 4 mL of 0.5 N NaOH and heated in boiling water for 15 min. After cooling to room temperature, 2 mL of 1 N HCl was added to neutralize the solution. The samples were centrifuged at 16,500 × *g* for 5 minutes, and the silica concentration in the supernatant was determined using the same silicate test kit. To account for differences in cell size between the strains, we measured their diameters using a Z2 Coulter counter. The amount of the insoluble silica per cell was divided by the square of the measured cell diameter.

### High-angle annular dark field scanning transmission electron microscopy and energy dispersive spectroscopy (HAADF STEM EDS)

For culturing *T. pseudonana* at either 10 µM Si or 330 µM Si (control), cells were first centrifuged at 3,000 × *g* for 10 min and resuspended in natural Mediterranean seawater containing 10 µM Si or 330 µM Si (control). This washing step was repeated twice. Subsequently, 1 mL of culture, adjusted to approximately 4 × 10⁵ cells mL⁻¹, was transferred into semi-permeable plastic inserts (‘ThinCert’, 12-well, 1 µm pore size; Greiner Bio-One). These inserts were floated on 400 mL reservoirs of Mediterranean seawater supplemented with Si to final concentrations of either 10 µM or 330 µM. Cells were cultured within the inserts for seven days. Cell concentrations were measured after four days using a Z2 Coulter counter. The cultures were then diluted with the corresponding medium to restore a density of approximately 4 × 10⁵ cells mL⁻¹ and allowed to grow for an additional three days. Valve extraction was subsequently performed using the sodium hypochlorite valve extraction method described above, and the cleaned frustules were mounted on TEM grids for imaging. Scanning Transmission Electron Microscope (S/TEM) experiments were performed on a Thermo Scientific Talos F200X microscope (200 kV), equipped with a Super-X large solid angle X-ray detector for EDS.

### Plunge freezing

Synchronized *T. pseudonana* cells were vitrified by plunge-freezing on glow-discharged 200 mesh copper R2/1 holey carbon film grids (Quantifoil Micro Tools GmbH, Grossloebichau, Germany). Using a Leica EM GP (Leica Microsystems GmbH, Wetzlar, Germany), 1 µl of filtered seawater was applied to the copper side to facilitate medium flow toward the blotting paper, followed by the application of 4 µL of cell suspension (4 × 10^6^ cells/ml) to the carbon side. Grids were blotted for 8 seconds from the back side of the grid and subsequently plunge-frozen in liquid ethane cooled by liquid nitrogen

### Cryo-FIB milling

Plunge-frozen grids containing cells were clipped into auto-grid rims (ThermoFisher Scientific) and transferred into the Zeiss Crossbeam 550 FIB/SEM dual beam microscope (Zeiss, Germany) using a Leica transfer station (VCM EM) equipped with a cryo stage. Prior to milling, grids were coated with organometallic platinum (trimethyl(methylcyclopentadienyl)platinum(IV)) by an in situ Gas Injection System (incorporated in the Zeiss chamber). Lamellae were milled in cells oriented along their girdle bands. Usually, lamella widths were typically limited to a few micrometers, spanning a single cell while retaining the mature valves at both edges of the lamella, so the lamellae are well supported. Stress-relief cuts were milled on each side of the lamellae. Lamellae were milled at a 16° angle relative to the grid plane. Rough milling to a thickness of 1 µm was performed in two steps using a 30 kV gallium ion beam at currents of 300 pA and 100 pA. In the final polishing step, lamellae were thinned to a final thickness of approximately 200 nm using a beam current of 50 pA.

### Cryo-electron tomography, volume reconstruction, and volume rendering

Cryo-electron tomography data were collected from ~30 cells. The tilt series was acquired using a Titan Krios G3i TEM (Thermo Fisher Scientific, Waltham, MA, USA), operating at 300 kV. Tilt series were recorded on a K3 direct detector (Gatan Inc., Pleasanton, CA, USA) installed behind a BioQuantum energy filter (Gatan Inc., Pleasanton, CA, USA), using a slit of 20 eV. All tilt series were acquired in counting mode at a nominal magnification of 33,000 × or 53,000 ×, corresponding to a physical pixel size of 0.26 nm or 0.16 nm, respectively. Data were collected using a dose-symmetric scheme starting from the lamella pre-tilt of −16° or 16° (dependent on grid orientation) and with 2° increments to acquire approximately 60 tilts per tomogram. Tilt series were recorded at a defocus range of 2-5 µm, using an objective aperture of 100 μm inserted. Automated low-dose acquisition was used throughout, resulting in a total accumulated dose of approximately 120 e⁻/Å² per tilt series.

Tomographic reconstructions were generated using either IMOD or AreTomo software. Segmentation was performed in Amira (Thermo Fisher Scientific, Waltham, MA, USA). Membranes were segmented using the membrane enhancement filter module, followed by manual refinement.

### Gap thickness measurements

Distances between the silica ribs were quantified using the measurement tool in AMIRA 3D software. For each valve, a corresponding 2D projection image was imported into the software, ensuring that the accurate pixel size calibration was maintained. Multiple distance measurements were taken at various locations across each valve to capture representative structural variation. The collected measurements of all distance values were subsequently exported for further quantitative analysis.

### Statistics and Reproducibility

All experiments were independently repeated with at least three biological samples, with similar results. Statistical analyses were performed using Microsoft Excel (XL). Comparisons among multiple groups were conducted using one-way ANOVA, and comparisons between two groups were performed using two-tailed Student’s *t*-tests. *P* values < 0.05 were considered statistically significant. For microscopy experiments, representative images from at least three biologically independent experiments are shown. Sample sizes are indicated in the Source file.

### Conclusion

Genetic control over biomineralization processes in general, and over diatom silicification specifically, is complex and multi-layered. Therefore, the specific functionality of the proteins that are involved in silica formation, the dAnk proteins being a recent addition to this family, is still limited. This study shows that the morphogenetic activity of the dAnk proteins is related to Si availability in the external medium and to the regulation of SDV formation. This puts the dAnk proteins as indirect regulators that synthesize various inputs to yield an output that determines pore formation in the silica valves. In order to holistically understand the factors regulating the silicification process, it will be important to aim for a multivariable framework that resembles other gene regulatory networks.

### Reporting summary

Further information on research design is available in the Nature Portfolio Reporting Summary linked to this article.

## Supplementary information

Supplementary Information

Reporting Summary

Transparent Peer Review file

## Data Availability

Cryo-electron tomography data are available through the Weizmann Institute of Science online library at https://doi.org/10.34933/0c43728a-2ab9-452c-9c37-4ed7d705dc68. Source data are provided with this paper. Any remaining information can be obtained from the corresponding author upon reasonable request.
