# Supplementary material for: The morphogenetic activity of dAnk genes in the diatom Thalassiosira pseudonana is sensitive to Si availability
Source: Commun Biol. 2026 Jun 29;9:1323. doi: 10.1038/s42003-026-10583-4 (PMC13652820; doi:10.1038/s42003-026-10583-4)
Supplement: Supplementary file 1 [file 42003_2026_10583_MOESM1_ESM.pdf]

Supplementary Information

The morphogenetic activity of dAnk genes in the diatom *Thalassiosira pseudonana* is sensitive to Si availability

Lior Aram, Neta Varsano, Olga Brontvein, Smadar Levin-Zaidman, Christoph Heintze & Assaf Gal

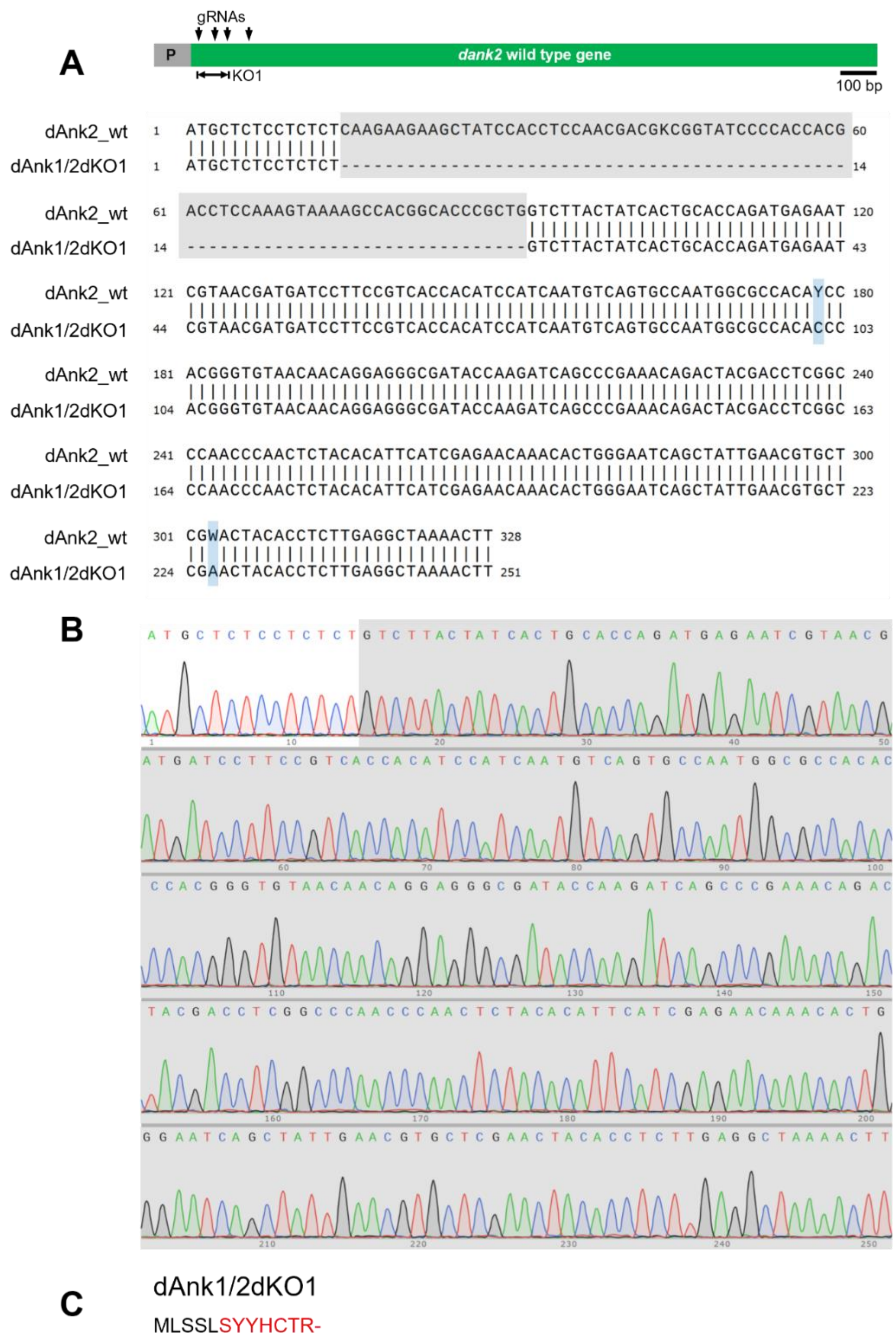

Figure S1. Sequence analysis of dAnk2-specific PCR products from the dAnk1 and dAnk2 double knockout (dAnk1/2dKO) strain. The dAnk1/2dKO1 strain was generated by introducing the dAnk2KO plasmid into the dAnk1KO1 single knockout strain. (A) The bar diagram shows the part of the promoter region (grey) and the entire protein coding region (green) of the dAnk2 gene. The binding sites for the guide RNAs (vertical arrows) and the deleted regions in the knockout strain (horizontal double arrows) are indicated. Underneath the bar diagram, a DNA sequence alignment of the dAnk2 gene from wild type

(top row) and dAnk1/2dKO1 (bottom row) is shown. The dAnk1/2dKO1 strain shows a 77 bp deletion in the coding region of the dAnk2 gene. Differences from the wild type sequence are highlighted in grey. The wild type dAnk2 gene has allelic variations in the dAnk2-specific PCR product that are highlighted in blue (Y=C or T, W=A or T). In the dAnk1/2dKO1 strain, only one of the alleles could be identified. Thus, the second dAnk2 allele might be completely lacking. (B) DNA sequence traces of the affected coding region of the dAnk2 gene in the dAnk1/2dKO1 strain are depicted. The DNA sequence begins with the start codon (ATG). Differences from the wild type sequence are highlighted in grey. (C) The amino acid sequence encoded by the mutated dAnk2 gene in the dAnk1/2dKO1 strain. Differences from the wild type sequence are highlighted in red. Premature termination of the amino acid sequence is depicted by '-'.

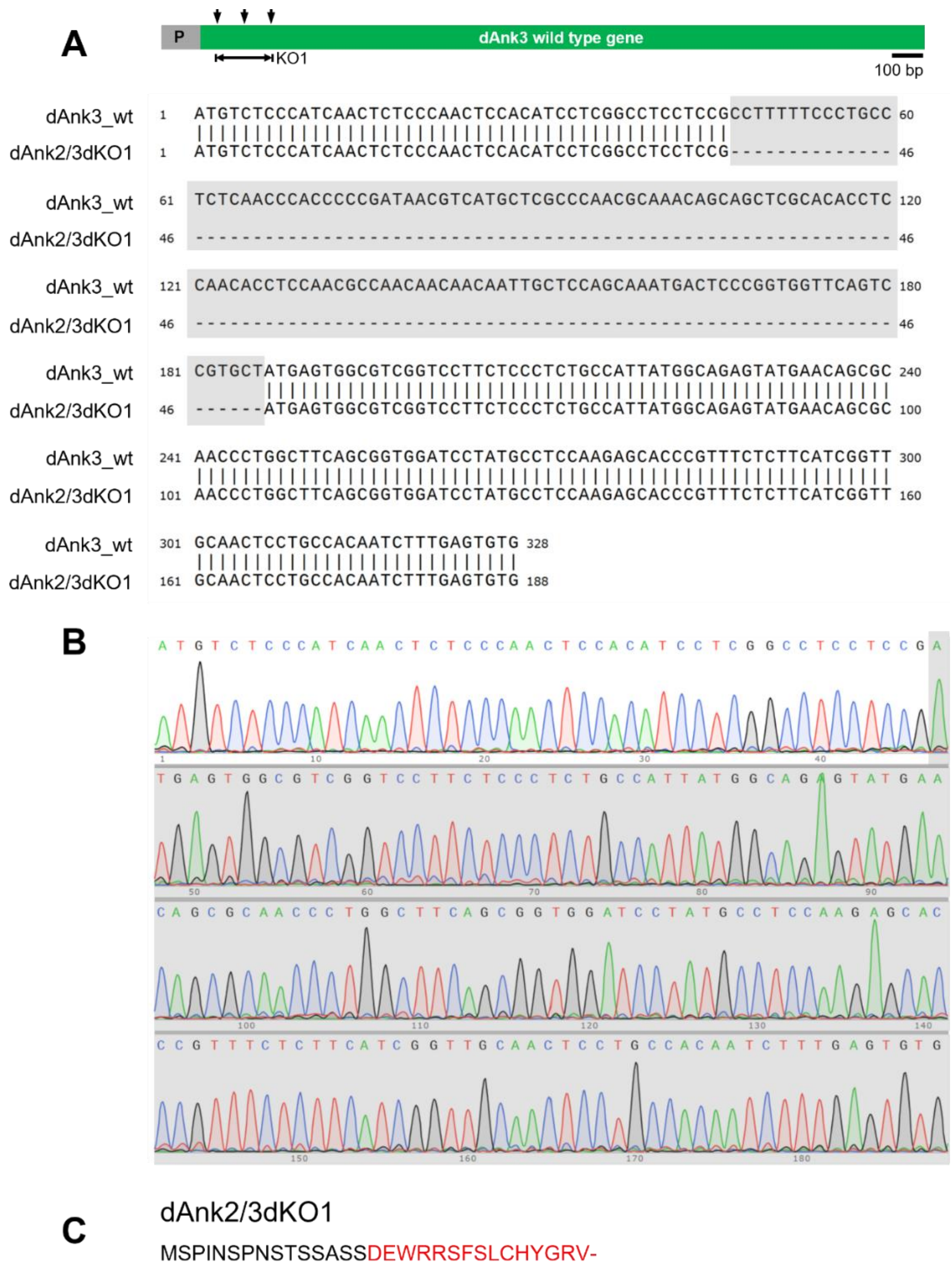

Figure S2. Sequence analysis of dAnk3-specific PCR products from the dAnk2 and dAnk3 double knockout (dAnk2/3dKO) strain. The dAnk2/3dKO1 strain was generated by introducing the dAnk3KO plasmid into the dAnk2KO1 single knockout strain. (A) The bar diagram shows the part of the promoter region (grey) and the entire protein coding region (green) of the dAnk3 gene. The binding sites for the guide RNAs (vertical arrows) and the deleted regions in the knockout strain (horizontal double arrows) are indicated. Underneath the bar diagram, a DNA sequence alignment of the dAnk3 gene from wild type

(top row) and dAnk2/3dKO1 (bottom row) is shown. The dAnk2/3dKO1 strain shows a 140 bp deletion in the coding region of the dank3 gene. Differences from the wild type sequence are highlighted in grey. (B) DNA sequence traces of the affected coding region of the dank3 gene in the dAnk2/3dKO1 strain are depicted. The DNA sequence begins with the start codon (ATG). Differences from the wild type sequence are highlighted in grey. The wild type dank3 gene has no allelic variation in the dank3-specific PCR product. Therefore, it is uncertain whether one dAnk3 allele is completely lacking in the dAnk2/3dKO strain or whether both dAnk3 alleles show the same mutation. (C) The amino acid sequence encoded by the mutated dAnk3 gene in the dAnk2/3dKO1 strain. Differences from the wild type sequence are highlighted in red. Premature termination of the amino acid sequence is depicted by '-'.

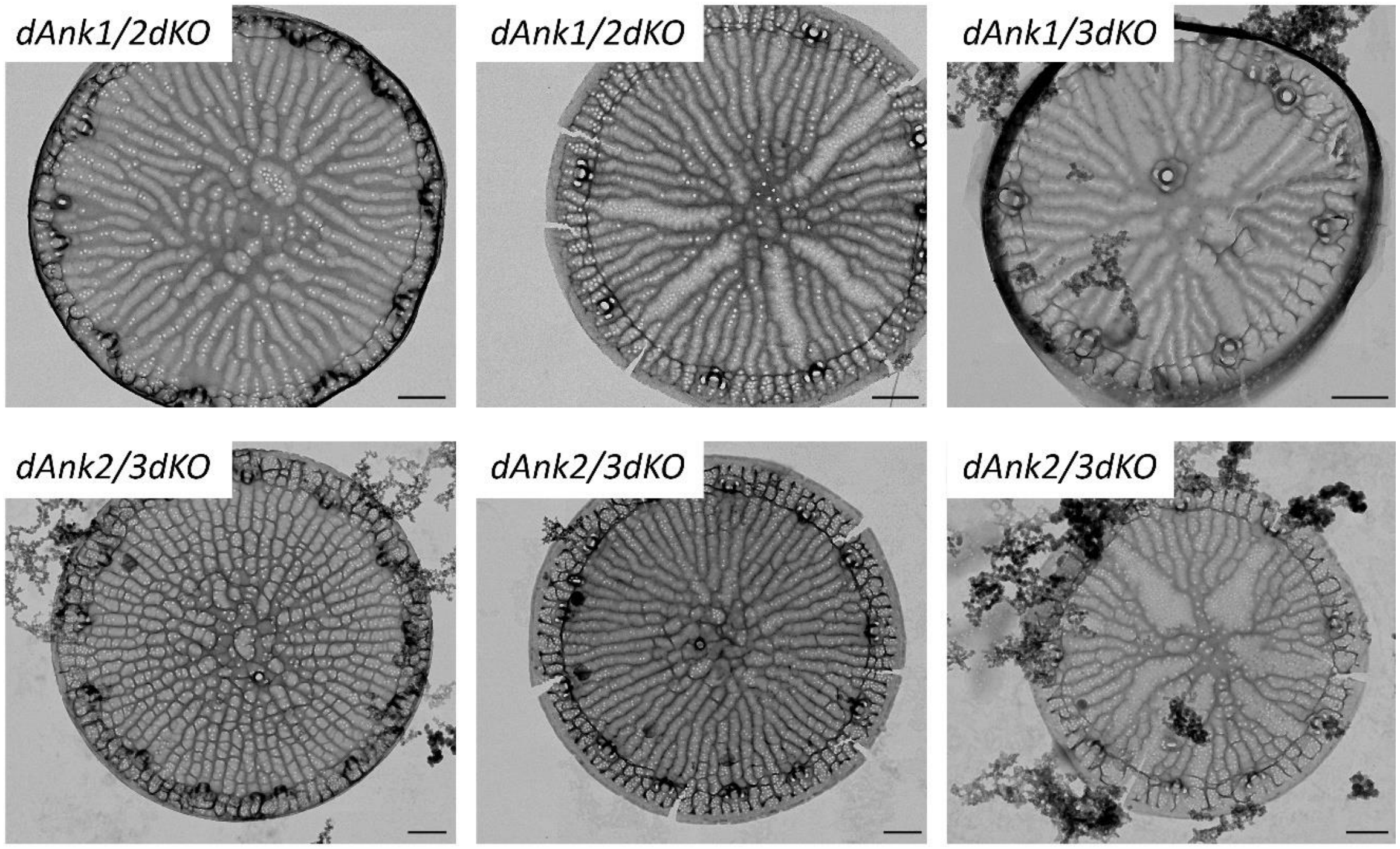

Figure S3. Double knockout valve phenotypes. TEM images of the extracted valves from the two new mutant strains, showing variability in their phenotypes. Scale bars: 500 nm.

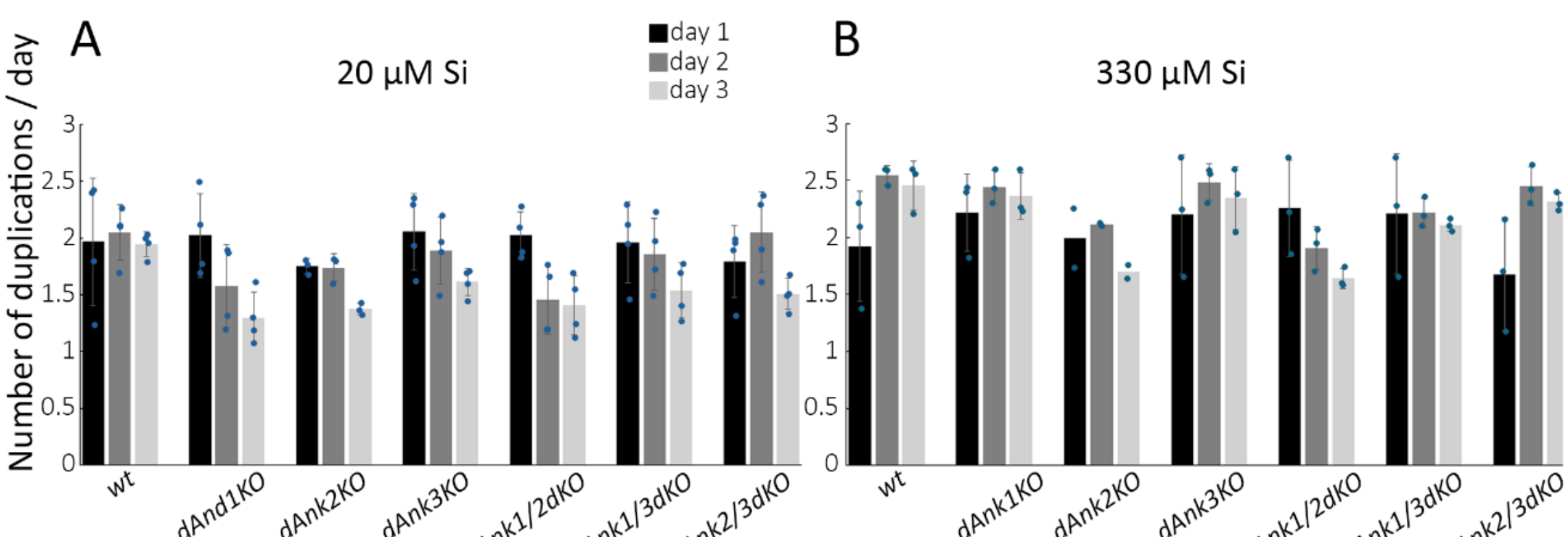

Figure S4. Culture growth in Si-deplete (20 µM Si) and Si-replete (330 µM Si) conditions. Data are presented as the average of three or four independent experiments ± standard deviation (except for the data of dAnk2dKO in 330 µM, which are presented as the average of two independent experiments). **(A, B)** Calculated duplication rates per day of the different mutants grown in 20 µM (A) or 330 µM (B) Si.

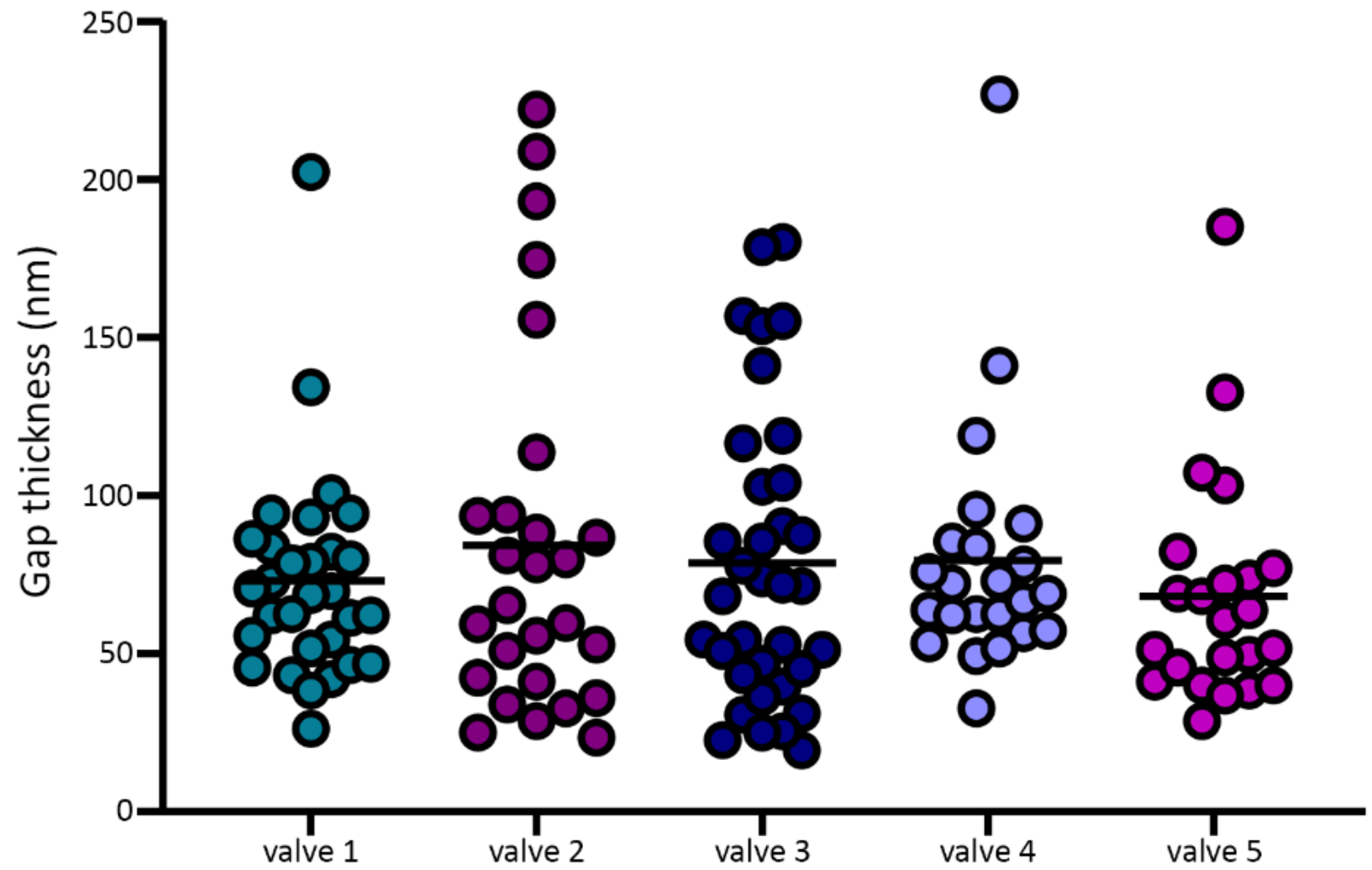

Figure S5. Gap thickness distribution. Gap thicknesses were measured in five *dAnk1/3dKO* valves, ranging from ~20 nm to ~220 nm.

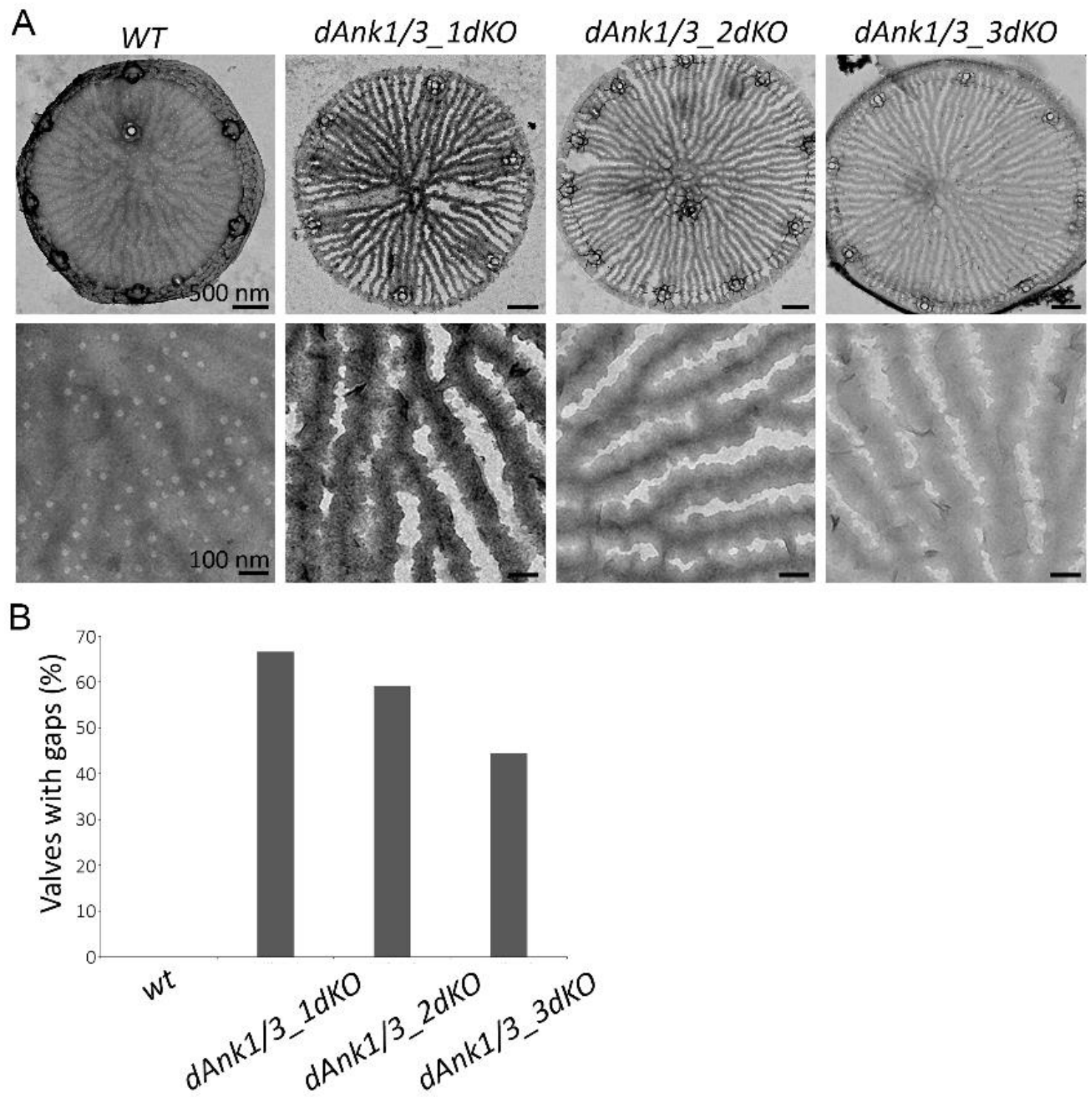

Figure S6. Two additional *dAnk1/3dKO* clones have the same elongated gap phenotype. (A) Elongated gap phenotype in two additional *dAnk1/3dKO* clones. Images in the second row show magnifications of the images in the first row. **(B)** Quantifying valves with elongated gaps of cultures grown in Si-deplete conditions (20 µM Si). The number of valves counted for each condition is in the Source data.

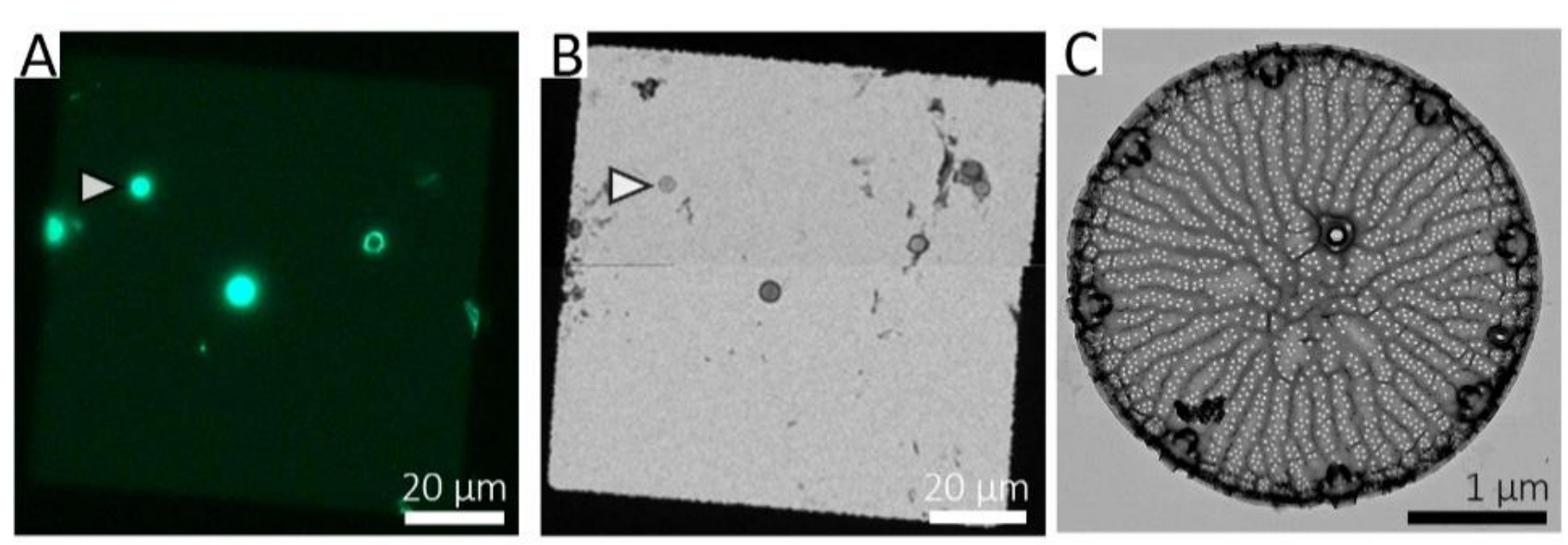

Figure S7. The correlative workflow. (A) PDMPO-positive valves indicate newly formed valves. **(B)** Corresponding electron micrograph of the same region shown in the fluorescence image in (A). **(C)** high magnification of PDMPO-positive valve, which is indicated by the white arrowhead in (B).

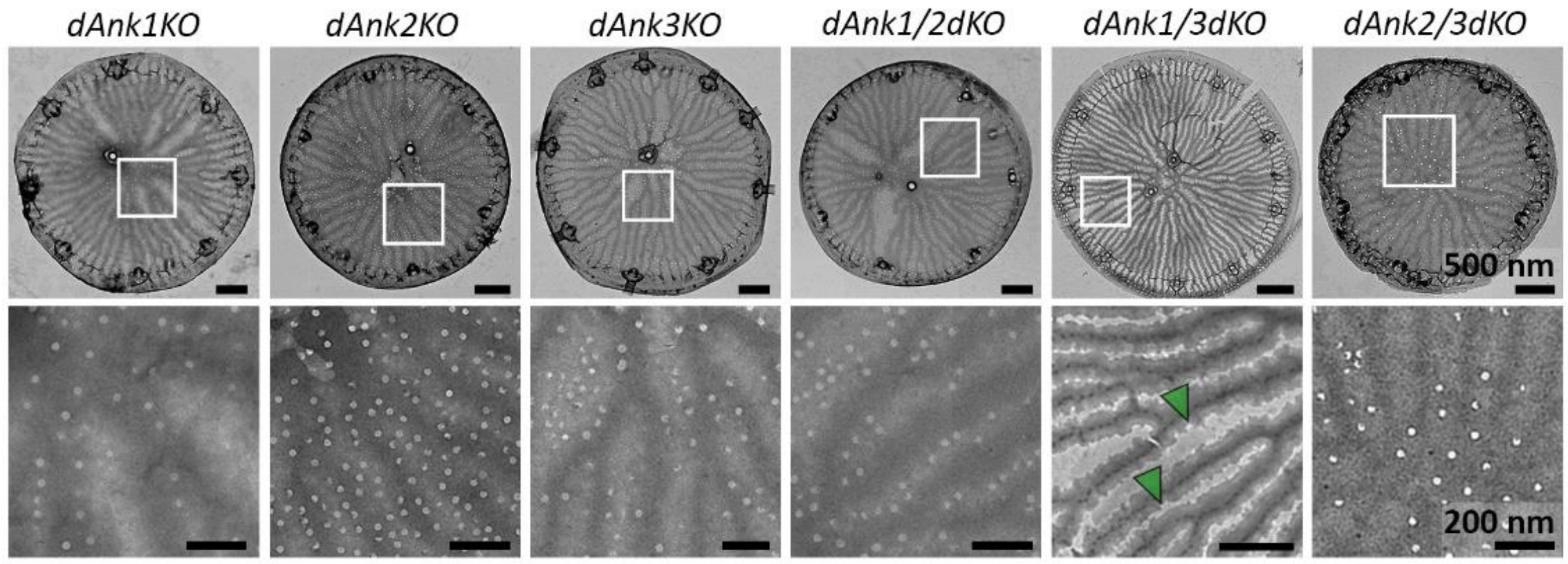

Figure S8. Elongated gaps under Si-starved conditions. TEM images of extracted valves of the six independent dAnk-KOs strains. Images in the second row show magnification of the indicated area. Green arrowheads – elongated gaps.

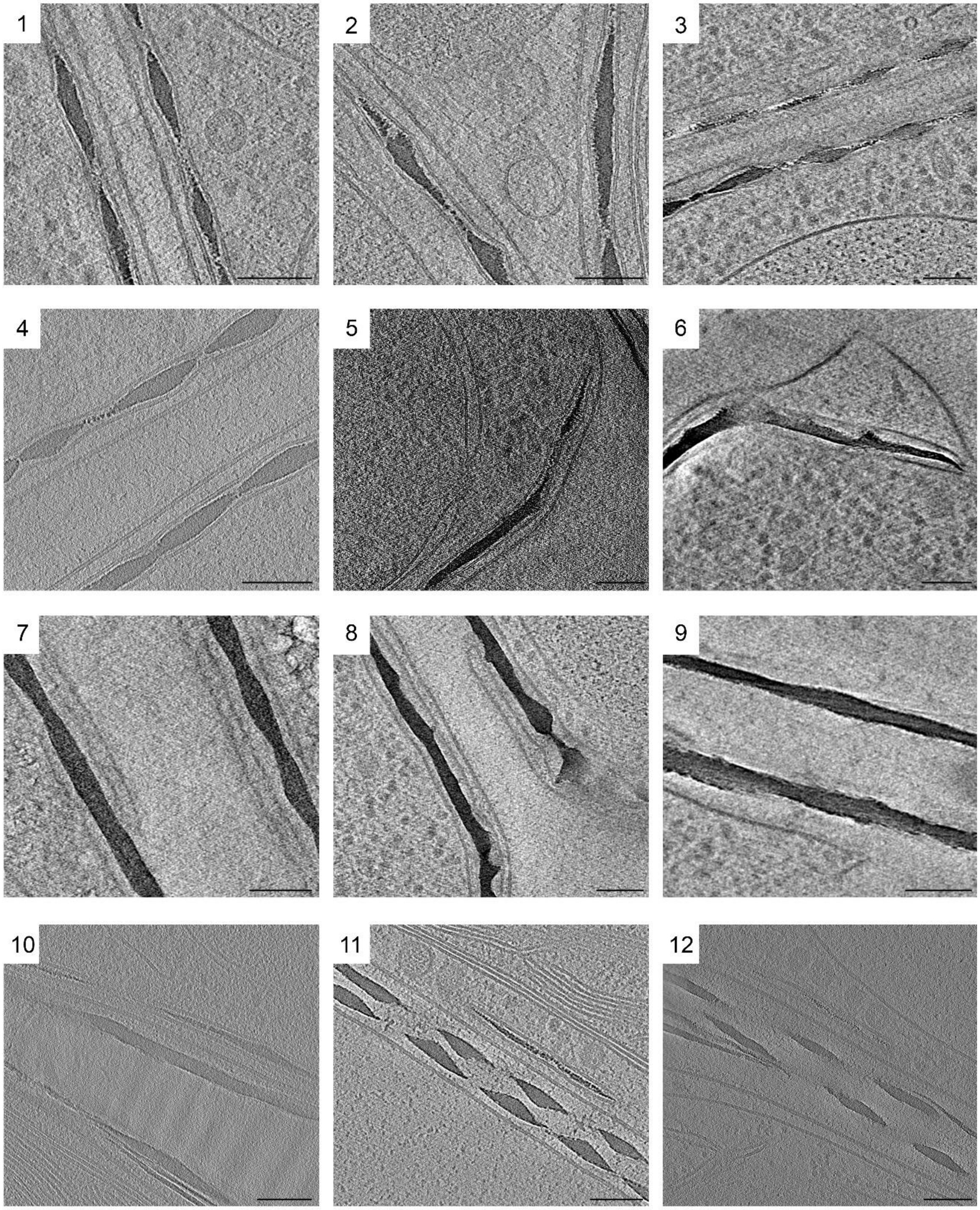

Figure S9a. Overview of the complete cryo-ET datasets. Plate 1 of 3. Representative slice from each of the 37 datasets. Scale bars, 100 nm.

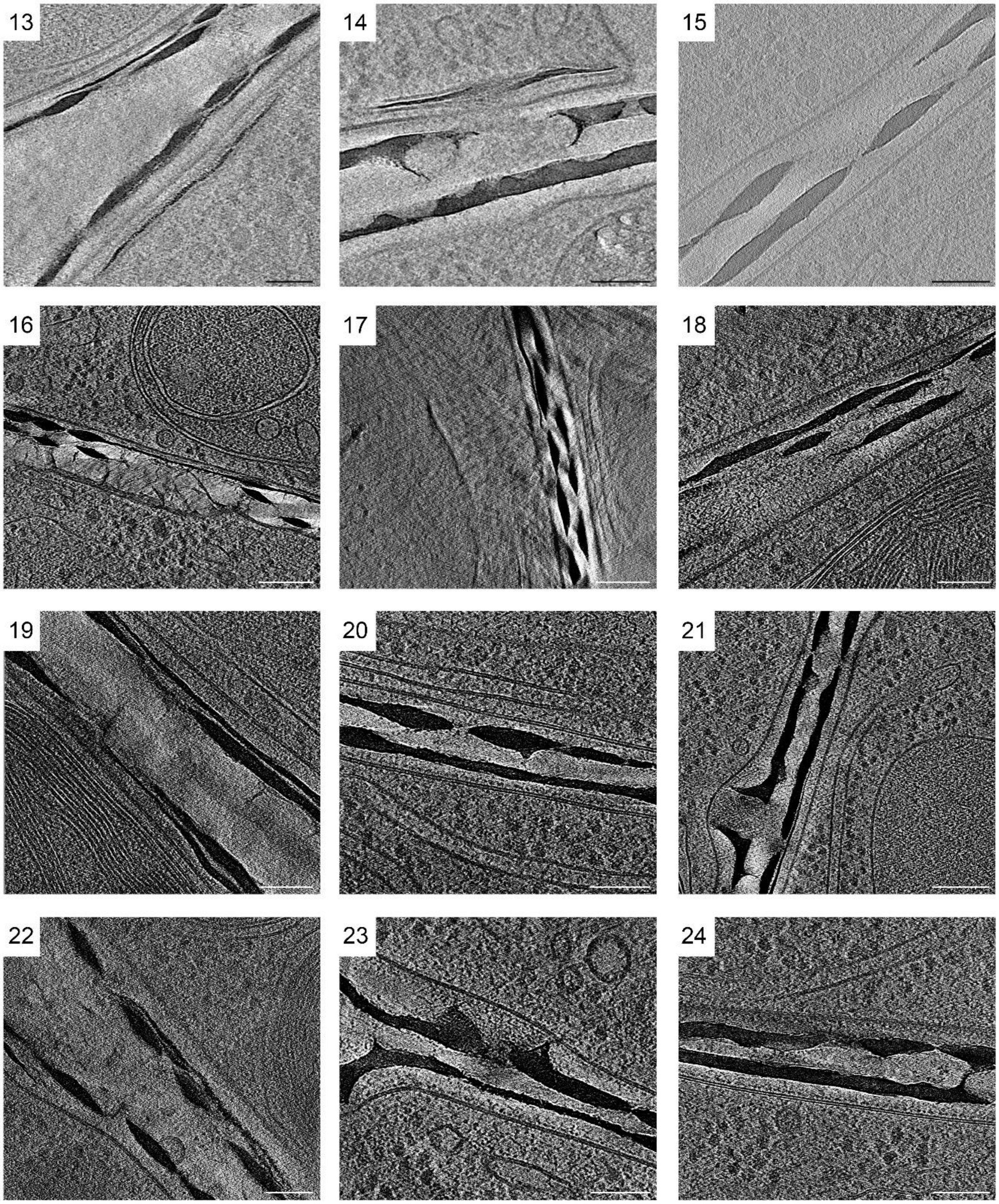

Figure S9b. Plate 2 out of 3.

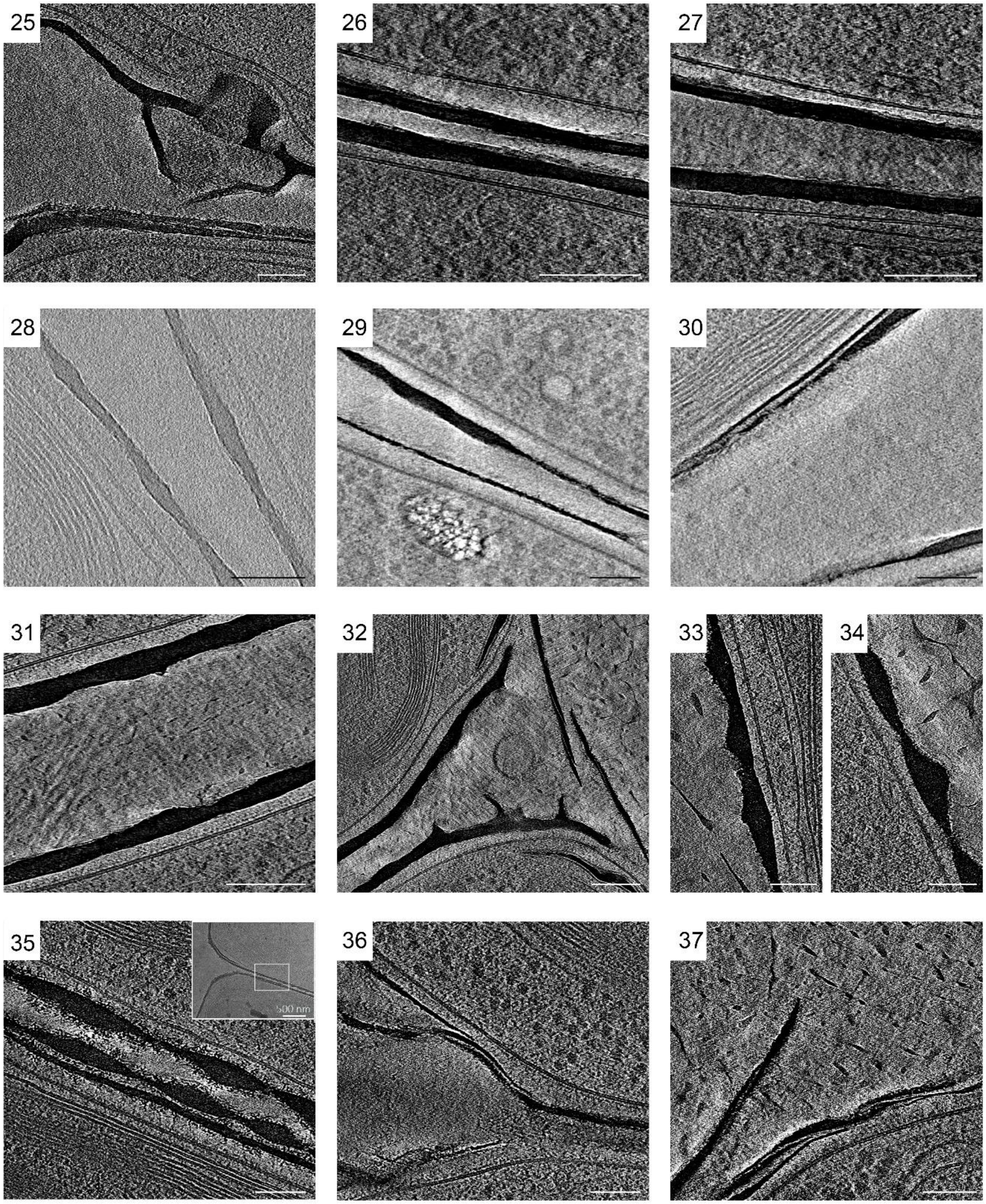

Figure S9c. Plate 3 out of 3.

Table S1

A representative long-term Si-limitation experiment showing cell concentrations, daily dilution factors, and the calculated percentage of original cells remaining in the culture on the day of the electron microscope sample preparations (grey column). Each row corresponds to a different *dAnk* knockout line grown in either 20 or 330 μM Si.

	0 hr	24 hr		48 hr		72 hr	
	x10^6 cells/ml	x10^6 cells/ml	dilution factor	x10^6 cells/ml	dilution factor	x10^6 cells/ml	% of original cells
wt_20	0.1	0.41	4	0.44	4	0.45	1.4
wt_330	0.1	0.51	5	0.50	5	0.46	0.9
dAnk1KO_20	0.1	0.34	3	0.29	3	0.20	5.5
dAnk1KO_330	0.1	0.67	5	0.59	5	0.56	0.7
dAnk2KO_20	0.1	0.29	3	0.29	3	0.25	4.5
dAnk2KO_330	0.1	0.58	5	0.42	4	0.33	1.5
dAnk3KO_20	0.1	0.39	4	0.36	3	0.33	2.5
dAnk3KO_330	0.1	0.58	5	0.56	4	0.58	0.9
dAnk12dKO_20	0.1	0.37	3	0.29	3	0.21	5.3
dAnk12dKO_330	0.1	0.56	5	0.37	3	0.37	1.8
dAnk13dKO_20	0.1	0.34	3	0.38	3	0.30	3.7
dAnk13dKO_330	0.1	0.59	5	0.45	4	0.51	1.0
dAnk23dKO_20	0.1	0.32	3	0.32	3	0.27	4.1
dAnk23dKO_330	0.1	0.54	5	0.47	4	0.56	0.9

Table S2

One-way ANOVA, testing the effect of Si concentration in the media on the duplication number of wt or dAnk1/3dKO cells, shows no difference between wt and dAnk1/3 after 24 hours (p=0.103) or 48 hours (p=0.165) in the two different Si concentrations.

One-way ANOVA testing the effect of Si concentration in the media on the insoluble silica of wt and dAnk1/3dKO cells grown in replete or deplete Si media shows no differences in insoluble silica (p=0.995).

One-way ANOVA testing the effect of Si concentration in the media on soluble silica in wt and dAnk1/3dKO cells grown in replete or deplete Si media shows a statistically significant difference within the group (p=0.01).

T-test to compare the differences in the soluble silica between the wt and the dAnk1/3dKO cells, which grew in replete and deplete Si media, shows significant differences between the replete and deplete Si media.

**Soluble silica comparisons**	**p-value**
wt330 – dAnk1/3dKO_330	0.798660004
wt20 - dAnk1/3dKO_20	0.560952385
wt330 - wt20	0.027937954*
dAnk1/3dKO_330 - dAnk1/3dKO_20	0.032250262*
