# Supplementary material for: The morphogenetic activity of dAnk genes in the diatom Thalassiosira pseudonana is sensitive to Si availability
Source: Commun Biol. 2026 Jun 29;9:1323. doi: 10.1038/s42003-026-10583-4 (PMC13652820; doi:10.1038/s42003-026-10583-4)
Supplement: Supplementary file 3 [file 42003_2026_10583_MOESM3_ESM.pdf]

nature portfolio

Peer Review File

The morphogenetic activity of dAnk genes in the diatom Thalassiosira pseudonana is sensitive to Si availability

Corresponding Author: Professor Assaf Gal

This file contains all reviewer reports in order by version, followed by all author rebuttals in order by version.

Version 0:

Reviewer comments:

Reviewer #1

(Remarks to the Author)
The manuscript presents novel and compelling results that provide valuable insight into the silicification process, particularly into a family of proteins involved in frustule formation. At present, silicification remains largely unexplored and poorly understood at the molecular level, which underscores the significance of the data presented. The authors investigated mutants of dAnk proteins from Thalassiosira pseudonana grown under varying environmental silicate concentration, enabling a more detailed assessment of the functional roles of this protein family. The use of cutting-edge techniques allowed for high-resolution morphological analyses of the mutants, revealing structural anomalies that would likely have remained undetected using less advanced techniques.
Given the significance of these data, I recommend improving the overall quality and fluency of the manuscript to better guide the reader through the various experimental setups. In particular, the use of different cultivation methods and environmental conditions would benefit from clearer explanation, as this complexity may be confusing to a non-specialist reader. In addition, the discussion appears somewhat underdeveloped in light of the breadth and importance of the results obtained and could be substantially strengthened to more fully contextualize and interpret the findings.
ABSTRACT
Line 19-26 - These lines briefly summarize the discussion of the results obtained and described in the previous lines. I find there's a significant gap and a lack of connection between the results and the discussion at this point. I understand that it's difficult to convey the results of a long study in just a few lines in an abstract, but they suggest a revisitation to clarify the intended message.
Line 24 The statement “When disrupted, silicification becomes sensitive to silicate availability, modifying pore patterns” is ambiguous. It is unclear whether “disrupted” refers to silicification in the mutants or to silicification under silicon starvation. If the former is intended, pores are already altered in the mutants under silicate-replete conditions, as shown in Figure 1. If the latter is meant, I did not find evidence in the manuscript for pore pattern modifications in the wild type under low silicate availability. I therefore suggest revising this sentence to clarify its meaning and to shift the emphasis toward the additional, and particularly significant, effects observed in the mutants under conditions of reduced silicon availability.
Line 26 “ This points to synergistic regulation of silicification, where the function of a specific protein is context-dependent.” This sentence points to an important finding that would benefit from deeper consideration and integration into the discussion.
INTRODUCTION
Overall, the introduction is well-written and presents the state of the art. I would highlight the working hypothesis, especially in line 66, where there's a gap between what's reported in the literature and the experiments conducted. It should be more consistent.
Line 69 – 73 I would better explain the different levels of experimental setups at this point rather than summarizing results. For instance, I suggest to clearly state in few lines what are the limitations treatments (since at least 3 different treatments were used), and the workflow that is then seen in the results.
MATERIAL AND METHODS
Line 46 supplemental materials : Do you have any reference about this concentration (< 3uM) blocking the cell cycle?
Line 84 analysis on soluble and unsoluble intracellular Si. I would add a short sentence explaining the importance of quantify both the pools, which is important for what deduction (this explanation can be put also in the main manuscript when describing results).
Line 92 I would define HAADF here in the supplementary material, just for reader which are not familiar with this technique
Line 93: Which is the rationale behind the choice of 10µM of silicate concentration, since the depletion experiments were done at 20µM and starvation at < 3µM Si.

RESULTS
Line 107 I should avoid the use of “normal”, I would rather use the term “replete”
Figure 2B for a better understanding of the figure, I would put the concentration of the 3 conditions (replete, deplete and starved)
Line 137 Is this “lower” statistically relevant?
Line 138 : a good point that should be addressed in the discussion
Line 146 “completely silicate deprived”. I should avoid this strong statement or better clarify the MM section line 47 (supplementary material). Indeed, if the “Si-free” medium is prepared from natural seawater, a small percentage of Si is still present, as you correctly stated before. Otherwise, if you prepared an artificial medium completely free from silicon, avoiding the use of glassware, you should clearly explain it in the MM section.
Line 175 Here I got a bit confused with the terms. You stated “we aimed to investigate the process of silica formation under Si limitation within the silica deposition vesicle”. In this step you use cells starved and then recovered with 10/20µM Si medium? Or with replete? Please clarify if the word “limitations” is used to explain the “starvation”.
Line 177 I should avoid the use of the term “normal”
Figure 6. When you talk about “low Si phenotypes” are you referring to mutants starved and replenished with replete Si medium (330µM)?
Line 219 – 222 This sentence is more a discussion
DISCUSSION
As explained before, I recommend a thorough review of the discussion and conclusions, which in my opinion are lacking in relation to the volume and importance of the data obtained.
I think the discussion lacks a clear comparison between wild type and mutants under the various Si-limiting conditions.
The discussion should be expanded on the results of the first set of experiments, in which it was observed that a growth-limiting silicon concentration did not affect the intracellular Si content, either soluble or insoluble, of either the mutants or the wild type.
The synergistic regulation of silicification by proteins and environmental condition is poorly addressed in the discussion

Reviewer #2

(Remarks to the Author)

This manuscript describes in detail the impact of mutations in the dAnk gene family on the morphology of the silica frustule of diatoms. The experiments are highly detailed, using a range of high-resolution microscopy approaches to allow the identification of novel phenotypes. The work provides novel insight into the cellular roles of the dAnk genes in frustule morphology.

The work is performed to a high standard and reveals new information about the cellular mechanisms of biomineralisation in diatoms. I have only a few minor comments.

Specific comments

line 110 – elongated gaps. This wasn’t clear to me immediately and I thought it referred to the distance between the ribs. Maybe clarify that this means ‘elongated gaps in the silica frustule’ and highlight that gaps mean that nanopores are absent.

Figure 2: As methods are only in Supplementary material, a brief description of Si-replete (330 uM), Si-limited (20 uM) etc would be helpful in the legend). Describe also the number of valves examined for 2B

Figure 3: I was confused with the units for insoluble silica fmol/cell um2 – is this correct? Please clarify and add replication (n=?) and define error bars

Line 165 The gaps lack Si, which is very clear from 4H, but then 4G shows EDS of the gap regions (pink/green boxes) with a clear Si signal. This should be clarified, is it simply the scale? I’m not sure what 4G is supposed to be demonstrating?

Line 211 Figure 6. it would be helpful to directly label the SDV and the silica layers in the images as these features are not immediately clear.

Line 257 As dAnk proteins are proposed to template nanopore formation the authors provide a nice explanation as to how the dAnk mutations could lead to the generation of gaps. The potential mechanisms through which dAnk mutations disrupt the SDV and lead to the formation of additional silica layers are less clear. Do the authors have any ideas on potential mechanisms?

Reviewer #3

(Remarks to the Author)
Review of Aram et al: The morphogenic activity of diatom dAnk genes is sensitive to Si availability

This manuscript builds on Heintze et al 2002 (PNAS: https://doi.org/10.1073/pnas.2211549119), in which some of the authors identified ankyrin containing proteins they named dAnks in diatoms. These proteins were shown to associate with

the silica deposition vesicle (SDV) and knock out mutants influenced the pore patterns of the silica. In the present manuscript, they extend this analysis to additional dAnk double mutants, examine the effects of silica starvation on the phenotypes, and characterize the details of valve formation in the mutants using cryo-FIB SEM. This is a worthwhile study that has delivered significant new insights into the dAnk mutants. For example, the authors show that the dAnk1/3 knock-out has gaps between silica ribs in silica-deplete conditions (which wild-type lacks), while under silica replete conditions, the silica has many less pores than wildtype. The unusual phenotypes in this mutant, involving abnormal SDVs and multiple silica layer revealed by cryo-FIB in some samples, are fascinating. In all, the study cements the dAnks as important regulators of silica structure between the ridges of the valves, but does not really add to our molecular understanding of how dAnks influence silica structure.

I have no major concerns about the manuscript, other than missing details about statistics, but there are some minor points I think should be addressed:

Line 17: Why “indirect”, and what is meant by this in the context?

Line 22 -23: I don’t think this conclusion is justified (see later)

Line 41:
https://www.pnas.org/doi/full/10.1073/pnas.2035131100 and
https://doi.org/10.1111/tpj.15765
Are missing from this list

Line 44: Not all are putative, some are demonstrated to transport Si: https://www.nature.com/articles/ncomms11926

Line 62: Insert “most” or “almost all” before “diatoms”, to account for P. tricornutum

Line 78: Insert after “generated” – “for this study” to improve clarity

Lines, 85, 92 and elsewhere: inconsistent use of the terms “cribrum pores” and “nanopores”. Use the same terminology throughout to make it clear you refer to the same thing.

Line 88 / 89: Reference figure

Paragraph including Line 90: A bit of rearrangement needed to make it clear to the reader what is new. Can you arrange it so that it is clear which mutants are new without the reader having to refer backwards?
Lines 112 – 115: Can you do better than “about” and “approximately”? For figure S5, do you have the equivalent data for wildtype?

Fig 2A: It would be helpful if the second row of part A was at the same scale for all images. I think there’s an argument for making this part of figure 1, to aid direct comparisons with silica replete conditions. In this case all the TEM images should be presented at the same scale. Arrowheads marking a single spot are perhaps not the best way to show the elongated gaps – something showing the length of the gap would be better.

Fig2: B is presented as a “statistical analysis”, yet I do not see any evidence of statistical analysis. Is each bar a single replicate culture?

Line 127 / 135 / 137 - 138: What statistical tests were used? What is n? What was the p-value?

Fig 3: State n + what the error bars represent.

Fig3B – are these values consistent with previous literature?

Fig3C and associated discussions: How do you reconcile this with the data on the thickening and thinning of parts of the valves in response to silica availability in the dAnk1/3 mutant that you show in other parts of the manuscript?

Line 145: Here you introduce silica starvation for the first time, yet it is also part of the experiments presented in Fig 2. The concept needs to be introduced earlier.

Line 165: How think a silica layer could be detected with the technique?

Line 177: change to “compared to Si replete conditions” for clarity

Line 190: Is it as simple as this? Fig 1 shows that the dAnk1/3 mutant has pores in some parts of the valve.

Discussion relating to Fig 6: Were sub abnormalities ever seen in wildtype? Were a comparable number of valves examined?

Line 237 – 239: Control of substrate availability seems to be just one possible explanation to me. For example, the dAnks

could regulate delivery of aspects of the SDV internal organic matrix (proteins, sugars …) which might then influence the affinity of the organic matrix between the ridges to silica precursors. If Si supply was the main effect, would you not expect rib silica to be affected and thus significant differences in Si mass per cell?

Lines 249 – 250: I don't see that cell to cell variability is really related to genetic redundancy.

Line 253: Could differing pool sizes of internal Si stores be a factor?

Line 264 – 265: What questions? Given previous studies, we expect diatoms with enough silica to make a valve to be able to divide.

Version 1:

Reviewer comments:

Reviewer #1

(Remarks to the Author)
The authors have satisfactorily addressed my previous concerns. The manuscript in this form presents compelling results regarding the role of dAnk proteins in frustule formation in Thalassiosira pseudonana.
I appreciate the authors' efforts to improve the overall quality, readability, and structure of the manuscript. The revised version is clearer, and the results are now better focused and more effectively communicated. In particular, the expanded discussion provides a more comprehensive interpretation of the findings and better contextualizes their importance.

At this stage, I recommend publication of the manuscript. I only suggest adding an appropriate reference to support the new sentence introduced in line 69.

Reviewer #2

(Remarks to the Author)
The authors have comprehensively addressed my comments on the initial manuscript. I have no further concerns.

Reviewer #3

(Remarks to the Author)
I am happy that all concerns have been addressed and the necessary clarifications to the manuscript have been made. I consider it suitable for publication in this revised form.

Open Access This Peer Review File is licensed under a Creative Commons Attribution 4.0 International License, which

permits use, sharing, adaptation, distribution and reproduction in any medium or format, as long as you give appropriate credit to the original author(s) and the source, provide a link to the Creative Commons license, and indicate if changes were made.
In cases where reviewers are anonymous, credit should be given to 'Anonymous Referee' and the source.
The images or other third party material in this Peer Review File are included in the article's Creative Commons license, unless indicated otherwise in a credit line to the material. If material is not included in the article's Creative Commons license and your intended use is not permitted by statutory regulation or exceeds the permitted use, you will need to obtain permission directly from the copyright holder.
To view a copy of this license, visit https://creativecommons.org/licenses/by/4.0/

Referee #1: Diatoms and environmental plant ecophysiology
Referee #2: Algal signaling and stress physiology
Referee #3: Algal biomineralization, proteomics, and bioinformatics

Reviewers' comments:

Reviewer #1 (Remarks to the Author):

The manuscript presents novel and compelling results that provide valuable insight into the silicification process, particularly into a family of proteins involved in frustule formation. At present, silicification remains largely unexplored and poorly understood at the molecular level, which underscores the significance of the data presented. The authors investigated mutants of dAnk proteins from Thalassiosira pseudonana grown under varying environmental silicate concentration, enabling a more detailed assessment of the functional roles of this protein family. The use of cutting-edge techniques allowed for high-resolution morphological analyses of the mutants, revealing structural anomalies that would likely have remained undetected using less advanced techniques.

Given the significance of these data, I recommend improving the overall quality and fluency of the manuscript to better guide the reader through the various experimental setups. In particular, the use of different cultivation methods and environmental conditions would benefit from clearer explanation, as this complexity may be confusing to a non-specialist reader. In addition, the discussion appears somewhat underdeveloped in light of the breadth and importance of the results obtained and could be substantially strengthened to more fully contextualize and interpret the findings.

We are grateful for the positive view of our work and its importance. We appreciate the detailed and thoughtful comments and did our best to modify the text in order to increase clarity depth.

ABSTRACT

Line 19-26 - These lines briefly summarize the discussion of the results obtained and described in the previous lines. I find there's a significant gap and a lack of connection between the results and the discussion at this point. I understand that it's difficult to convey the results of a long study in just a few lines in an abstract, but they suggest a revisitation to clarify the intended message.

We have added two sentences to provide more information on the actual results and to connect the information on the results and conclusions. The revised text is in lines 19-24: "Using various microscopy techniques, we show that the phenotype of some

mutants is variable, ranging from large gaps in the silica under silicate-deplete conditions to pore-less silica coverage under silicate-replete conditions. This gap phenotype is the result of incomplete silicification process within the cell. In situ cryo-electron tomography shows that not only is morphogenesis affected, but also the regulation of the silica deposition vesicle is altered. This is manifested in the observation of abnormal silica deposition vesicles that are not synchronized with the cell cycle."

Line 24 The statement "When disrupted, silicification becomes sensitive to silicate availability, modifying pore patterns" is ambiguous. It is unclear whether "disrupted" refers to silicification in the mutants or to silicification under silicon starvation. If the former is intended, pores are already altered in the mutants under silicate-replete conditions, as shown in Figure 1. If the latter is meant, I did not find evidence in the manuscript for pore pattern modifications in the wild type under low silicate availability. I therefore suggest revising this sentence to clarify its meaning and to shift the emphasis toward the additional, and particularly significant, effects observed in the mutants under conditions of reduced silicon availability.

The Reviewer is absolutely correct; we intend to say that the mutants are sensitive to silicate. The sentence was revised to eliminate ambiguity. Lines 25-27: "These results point to an additional role of the dAnk proteins, where they are involved in the regulation of intracellular silicification, and when their expression is disrupted, silicification becomes sensitive to silicate availability."

Line 26 " This points to synergistic regulation of silicification, where the function of a specific protein is context-dependent." This sentence points to an important finding that would benefit from deeper consideration and integration into the discussion.

Thank you. We expanded on this point in the revised discussion.

INTRODUCTION

Overall, the introduction is well-written and presents the state of the art. I would highlight the working hypothesis, especially in line 66, where there's a gap between what's reported in the literature and the experiments conducted. It should be more consistent.

We have added a sentence at the end of this paragraph to stress this working hypothesis of diatom silicification. Lines 67-69: "In wild-type cells, silica morphology is not affected by limiting Si conditions, and once Si is above the minimal threshold, the cells silicify in accordance with the same regulated morphology."

Line 69 – 73 I would better explain the different levels of experimental setups at this point rather than summarizing results. For instance, I suggest to clearly state in few lines

what are the limitations treatments (since at least 3 different treatments were used), and the workflow that is then seen in the results.

At this point, we have a different view from the reviewer. We think it is better to finish the introduction with a glimpse of the results, so an interested reader will carry on reading and understand the methodology along with the full description of the results.

MATERIAL AND METHODS

Line 46 supplemental materials : Do you have any reference about this concentration (< 3uM) blocking the cell cycle?

Arrest of the cell cycle was experimentally validated by monitoring that there was no substantial increase in cell number using this medium. We added the following sentence to the section, now in lines 47-49: "Cell cycle arrest was confirmed as prolonged growth in this medium yielded less than 20% increase in cell counts."

Line 84 analysis on soluble and unsoluble intracellular Si. I would add a short sentence explaining the importance of quantify both the pools, which is important for what deduction (this explanation can be put also in the main manuscript when describing results).

We elaborate on the aim of quantification in the revised sentence. Lines 80-82: "The soluble and insoluble silica contents in wild-type and dAnk1/3dKO cells were quantified following an established protocol, in order to investigate if there is a difference between the various strains."

Line 92 I would define HAADF here in the supplementary material, just for reader which are not familiar with this technique.

The full wording is now provided in the revised section.

Line 93: Which is the rationale behind the choice of 10µM of silicate concentration, since the depletion experiments were done at 20µM and starvation at < 3µM Si.

While developing the project we experimented with several Si-deplete growth conditions. Actually, the initial one was as described for the HAADF-STEM experiments (10µM Si and cells in inserts), but since growth was too sensitive to sudden collapsing of the culture, we report the rest of the experiments in a second procedure (20µM Si and daily dilutions).

RESULTS

Line 107 I should avoid the use of "normal", I would rather use the term "replete".

Corrected to 'Si-replete'. Now in line 113.

Figure 2B for a better understanding of the figure, I would put the concentration of the 3 conditions (replete, deplete and starved).

This addition was made to Fig. 2 B, which now includes also Si concentrations.

Line 137 Is this "lower" statistically relevant?

Statistical information is now added to the Figure caption and in Table S2 in the Supplementary file.

Line 138 : a good point that should be addressed in the discussion.

Thank you. This point is included in the revised Discussion (see below).

Line 146 "completely silicate deprived". I should avoid this strong statement or better clarify the MM section line 47 (supplementary material). Indeed, if the "Si-free" medium is prepared from natural seawater, a small percentage of Si is still present, as you correctly stated before. Otherwise, if you prepared an artificial medium completely free from silicon, avoiding the use of glassware, you should clearly explain it in the MM section.

The Reviewer is correct. We changed the phrasing to "cells were transferred to a silicate-deprived medium in which cell-cycle is arrested for 16 hours." Now in lines 165-166.

Line 175 Here I got a bit confused with the terms. You stated "we aimed to investigate the process of silica formation under Si limitation within the silica deposition vesicle". In this step you use cells starved and then recovered with 10/20µM Si medium? Or with replete? Please clarify if the word "limitations" is used to explain the "starvation".

The Reviewer is 100% correct. We used the wrong terminology as the cryoET experiments were done on cells recovering from Si starvation. The revised sentence in lines 196-197: "In the next step, we aimed to investigate the process of silica formation within the silica deposition vesicle (SDV) of cells that recover from Si starvation, to evaluate what are the structural differences compared to the process at Si-replete conditions."

Line 177 I should avoid the use of the term "normal"

Agreed. We now use "Si-replete conditions". See full sentence in the previous comment.

Figure 6. When you talk about "low Si phenotypes" are you referring to mutants starved and replenished with replete Si medium (330µM)?

Yes, as clarified above, all cryoET experiments were conducted after Si starvation and replenishment.

Line 219 – 222 This sentence is more a discussion

Indeed, this is per our writing style where after each set of experimental results we summarize the main take home message.

DISCUSSION

As explained before, I recommend a thorough review of the discussion and conclusions, which in my opinion are lacking in relation to the volume and importance of the data obtained.

We have expanded every point in the Discussion in order to be in line with the nature of the comments that we received from all Reviewers.

I think the discussion lacks a clear comparison between wild type and mutants under the various Si-limiting conditions.

This comparison is broken into three aspects now, in the first three paragraphs of the Discussion.

The discussion should be expanded on the results of the first set of experiments, in which it was observed that a growth-limiting silicon concentration did not affect the intracellular Si content, either soluble or insoluble, of either the mutants or the wild type.

This is now discussed in lines 260-264.

The synergistic regulation of silicification by proteins and environmental condition is poorly addressed in the discussion

We hope the Discussion is improved. We note that no clear mechanism is currently available as we state in lines 296-298: “Nevertheless, currently there is not enough information to substantiate a comprehensive mechanism that will link all the observations, and further genetic and structural studies are needed.”

Reviewer #2 (Remarks to the Author):

This manuscript describes in detail the impact of mutations in the dAnk gene family on the morphology of the silica frustule of diatoms. The experiments are highly detailed, using a range of high-resolution microscopy approaches to allow the identification of novel phenotypes. The work provides novel insight into the cellular roles of the dAnk genes in frustule morphology.
The work is performed to a high standard and reveals new information about the cellular mechanisms of biomineralisation in diatoms. I have only a few minor comments.

We very much appreciate the positive evaluation of our work.

Specific comments
line 110 – elongated gaps. This wasn't clear to me immediately and I thought it referred to the distance between the ribs. Maybe clarify that this means 'elongated gaps in the silica frustule' and highlight that gaps mean that nanopores are absent.

We agree that it would be good to annotate in the images what do we mean by 'elongated gaps'. Panel A in Fig. 2 was modified to include such visual guidance in the rm of blue annotations.

Figure 2: As methods are only in Supplementary material, a brief description of Si-replete (330 uM), Si-limited (20 uM) etc would be helpful in the legend). Describe also the number of valves examined for 2B.

This comment is in line with a similar from Reviewer 1 and the legend and number of valves are now provided in the source data file.

Figure 3: I was confused with the units for insoluble silica fmol/cell um2 – is this correct? Please clarify and add replication (n=?) and define error bars.

Yes, the units are correct since we divided the measured amount of silica per cell by the area of the valves in order to account for different cell sizes between the strains. We explain this calculation in more detail in the revised Methods section, lines 93-96. Statistical information was also improved.

Line 165 The gaps lack Si, which is very clear from 4H, but then 4G shows EDS of the gap regions (pink/green boxes) with a clear Si signal. This should be clarified, is it simply the scale? I'm not sure what 4G is supposed to be demonstrating?

Panel G includes two spectra, the green is from a dAnk1/3dKO valve where Si is detected between the ribs, and the pink is from the mutant where no Si is detected from the gap. We revised the text in the caption in order to clarify this: lines 190-191: "(G) EDS

spectrum of the green (D – where thin silica is present) and pink (F – an empty gap) areas in D and F.”

Line 211 Figure 6. it would be helpful to directly label the SDV and the silica layers in the images as these features are not immediately clear.

We are sorry, but we couldn’t understand what exactly the Reviewer means. In Fig. 6 all panels contain color coded annotations for the SDV and silica layers. We note that the caption lacked information about the presence of two cells in the image so we added the following sentence in lines 248-250: “Each dataset contains regions from two daughter cells, in the middle and right columns, surface representations that relate only to the lower cell in each dataset are presented for the segmented silica, SDV membranes, and plasma membrane.”

Line 257 As dAnk proteins are proposed to template nanopore formation the authors provide a nice explanation as to how the dAnk mutations could lead to the generation of gaps. The potential mechanisms through which dAnk mutations disrupt the SDV and lead to the formation of additional silica layers are less clear. Do the authors have any ideas on potential mechanisms?

We wish we could provide some evidence-based model or hypothesis, but currently we can just generally relate these observations as presented in more detail in the expanded Discussion, and specifically in lines 296-298 “Nevertheless, currently there is not enough information to substantiate a comprehensive mechanism that will link all the observations, and further genetic and structural studies are needed.”

Reviewer #3 (Remarks to the Author):

Review of Aram et al: The morphogenic activity of diatom dAnk genes is sensitive to Si availability
This manuscript builds on Heintze et al 2002 (PNAS: https://doi.org/10.1073/pnas.2211549119), in which some of the authors identified ankyrin containing proteins they named dAnks in diatoms. These proteins were shown to associate with the silica deposition vesicle (SDV) and knock out mutants influenced the pore patterns of the silica. In the present manuscript, they extend this analysis to additional dAnk double mutants, examine the effects of silica starvation on the phenotypes, and characterize the details of valve formation in the mutants using cryo-FIB SEM. This is a worthwhile study that has delivered significant new insights into the dAnk mutants. For example, the authors show that the dAnk1/3 knock-out has gaps between silica ribs in silica-deplete conditions (which wild-type lacks), while under silica replete conditions, the silica has many less pores than wildtype. The unusual phenotypes in this mutant, involving abnormal SDVs and multiple silica layer revealed by cryo-FIB in some samples, are fascinating. In all, the study cements the dAnks as important regulators of silica structure between the ridges of the valves, but does not really add to our molecular understanding of how dAnks influence silica structure.

Thank you for your efforts in evaluating and commenting on the paper. We appreciate the positive and constructive feedback.

I have no major concerns about the manuscript, other than missing details about statistics, but there are some minor points I think should be addressed:
Line 17: Why "indirect", and what is meant by this in the context?

'Indirect' means that no physical interaction occurs between the dAnk proteins and the forming silica. For clarity, we removed the two uses of 'indirect' from this sentence.

Line 22 -23: I don't think this conclusion is justified (see later)

We agree that the conclusion was not accurately phrased. It was replaced with the following statement, lines25-27: "These results point to an additional role of the dAnk proteins, where they are involved in the regulation of intracellular silicification, and when their expression is disrupted, silicification becomes sensitive to silicate availability."

Line 41:
https://www.pnas.org/doi/full/10.1073/pnas.2035131100 and

https://doi.org/10.1111/tpj.15765

Are missing from this list

Thank you. These references were added.

Line 44: Not all are putative, some are demonstrated to transport Si: https://www.nature.com/articles/ncomms11926

Thank you for suggesting this work, but since it demonstrated Si transport activity in vitro and not in diatoms, we are hesitant to consider it as clear evidence for functionality in vivo. We are more critical than what might be expected regarding this point since unpublished knockout experiments of these genes in vivo do not result in a significant phenotype (in line with previous published knock down experiments by the Hildebrand group).

Line 62: Insert "most" or "almost all" before "diatoms", to account for P. tricornutum

We inserted 'almost all' in line 63 as suggested.

Line 78: Insert after "generated" – "for this study" to improve clarity

Corrected as suggested. Now in line 82.

Lines, 85, 92 and elsewhere: inconsistent use of the terms "cribrum pores" and "nanopores". Use the same terminology throughout to make it clear you refer to the same thing.

Only the first use of 'cribrum pore' was left and the others (next sentence and Fig. 1 caption) modified to 'nanopores'.

Line 88 / 89: Reference figure

A reference to Fig. 1 is now included in line 94.

Paragraph including Line 90: A bit of rearrangement needed to make it clear to the reader what is new. Can you arrange it so that it is clear which mutants are new without the reader having to refer backwards?

We understand the point. In order to clarify which phenotypes are already established we added in line 90: "The previously reported mutant strains demonstrate their identified phenotypes,25 in the dAnk1KO strain…". Also in the rest of the paragraph we name the 'new' and 'reported' strains.

Lines 112 – 115: Can you do better than "about" and "approximately"?

Of course. The exact numbers are presented in Fig. 2 B and in the raw data associated. We realize that we did not include a reference to the data in the text, which is now corrected and the reader is referred to the data in line 120.

For figure S5, do you have the equivalent data for wildtype?

Since there were too few gaps in the wt we did not analyze their statistics.

Fig 2A: It would be helpful if the second row of part A was at the same scale for all images. I think there's an argument for making this part of figure 1, to aid direct comparisons with silica replete conditions. In this case all the TEM images should be presented at the same scale. Arrowheads marking a single spot are perhaps not the best way to show the elongated gaps – something showing the length of the gap would be better.

We have revised Fig. 2 A so now each row has a consistent scale, and the annotations were replaced with continuous coloring of gaps. We understand that combining Figs. 1 and 2 can help with comparisons, but it will also 'dilute' the new findings with previously published phenotypes, so we prefer to keep it separate.

Fig2: B is presented as a "statistical analysis", yet I do not see any evidence of statistical analysis. Is each bar a single replicate culture?

We changed the wording to just 'Analysis' since Fig. 2 B is simply the results of counting valves with gaps across all imaged valves. The raw data is provided.

Line 127 / 135 / 137 - 138: What statistical tests were used? What is n? What was the p-value?

These details are now presented in the caption of Fig. 3.

Fig 3: State n + what the error bars represent.

Added to the caption.

Fig3B – are these values consistent with previous literature?

Yes, the values of the control (wt at silica-replete conditions) are similar to what was reported in Refence 27, Fig. 5A.

Fig3C and associated discussions: How do you reconcile this with the data on the thickening and thinning of parts of the valves in response to silica availability in the dAnk1/3 mutant that you show in other parts of the manuscript?

This is a good point, which suggests that the extent of gap formation does not significantly change the overall cellular Si budget. We can speculate that the inter-rib silica is initially thin and contributes only a minor part, and that its partial loss is compensated by more silica in the ribs. We mention it now in lines 146-149: “These findings suggest that dAnk1/3dKO does not affect silica uptake, production, or cell viability, even in Si-deplete conditions that affect the level of intracellular soluble Si. This means that silica absent from the mutant gaps is compensated in other elements, such as thicker ribs.”

Line 145: Here you introduce silica starvation for the first time, yet it is also part of the experiments presented in Fig 2. The concept needs to be introduced earlier.
It is true that Fig. 2 B contains analysis described later. In the caption we include “(Si-starved experiments are presented later in the text)”

Line 165: How think a silica layer could be detected with the technique?

The minimal detection limit of EDS is dependent on various sample properties, but can be down to single atomic layers, so we think it is sensitive enough for this conclusion.

Line 177: change to “compared to Si replete conditions” for clarity

Corrected.

Line 190: Is it as simple as this? Fig 1 shows that the dAnk1/3 mutant has pores in some parts of the valve.

We revised the sentence to a more accurate phrasing. Lines 210-212: “…mature valves that formed before Si-starvation were characterized by the absence of nanopores (Fig. 5H), same phenotype that was observed by imaging mature extracted valves.”

Discussion relating to Fig 6: Were sub abnormalities ever seen in wildtype? Were a comparable number of valves examined?

Yes. The wt was extensively studied and we re-examined all the data related to our previous publication (Aram et al. Nat. Commun. 2024). Abnormal SDVs and layered silica were never observed in these datasets.

Line 237 – 239: Control of substrate availability seems to be just one possible explanation to me. For example, the dAnks could regulate delivery of aspects of the SDV internal organic matrix (proteins, sugars …) which might then influence the affinity of the organic matrix between the ridges to silica precursors. If Si supply was the main effect, would you not expect rib silica to be affected and thus significant differences in Si mass per cell?

The most likely scenario is a combination of the two effects. The dAnk proteins are affected by Si availability, but not directly since the effect is limited to the inter-rib space. We modified this part of the discussion. Lines 261-264: “It is evident that the altered pore phenotype is dependent of other factors such as silicate availability, pointing to a role for a possible relation of the dAnk proteins to regulation of silica transport into the SDV, even though they do not directly alter cellular Si content and viability.”

Lines 249 – 250: I don’t see that cell to cell variability is really related to genetic redundancy.

We think that redundancy in the dAnk proteins might be related to the fact that some cells have distinct phenotype and others do not. This phenotype is not a complete loss-of-function as the role is indirect.

Line 253: Could differing pool sizes of internal Si stores be a factor?

In principle yes, but the current level of understanding intracellular Si pool is too ambiguous to directly speculate its involvement.

Line 264 – 265: What questions? Given previous studies, we expect diatoms with enough silica to make a valve to be able to divide.

WT cells will synchronize cell cycle with silicification. The abnormal SDVs in the mutant raise questions if this coupling is compromised in the mutant lines.

Reviewer #1 (Remarks to the Author):

The authors have satisfactorily addressed my previous concerns. The manuscript in this form presents compelling results regarding the role of dAnk proteins in frustule formation in Thalassiosira pseudonana.

I appreciate the authors' efforts to improve the overall quality, readability, and structure of the manuscript. The revised version is clearer, and the results are now better focused and more effectively communicated. In particular, the expanded discussion provides a more comprehensive interpretation of the findings and better contextualizes their importance.

At this stage, I recommend publication of the manuscript. I only suggest adding an appropriate reference to support the new sentence introduced in line 69.

We are grateful for the positive view of our work. We added a reference at line 69.
